# Diet-Related Health Inequalities in High-Income Countries: A Scoping Review of Observational Studies

**DOI:** 10.1016/j.advnut.2025.100439

**Published:** 2025-05-05

**Authors:** Elena Carrillo-Alvarez, Rosa Rifà-Ros, Blanca Salinas-Roca, Lluís Costa-Tutusaus, Mafalda Lamas, Míriam Rodriguez-Monforte

**Affiliations:** 1 Universitat Ramon Llull, Blanquerna School of Health Sciences, Global Research on Wellbeing Research Group, Barcelona, Spain; 2 Independent Researcher, Lisbon, Portugal

**Keywords:** diet, inequalities, high-income countries, social determinants of health

## Abstract

Diet-related health inequalities are a persistent public health challenge in high-income countries, disproportionately affecting socially and economically disadvantaged populations. This study aims to map the existing evidence on diet-related health inequalities in high-income countries through a scoping review of observational studies, identifying populations most affected and key dietary outcomes across social determinants of health. We conducted a systematic search of MEDLINE, Web of Science, Scopus, and Embase for observational studies published between January 2011 and March 2021. Eligible studies assessed diet-related health outcomes stratified by ≥1 Place of Residence, Race/Ethnicity, Occupation, Gender/Sex, Religion, Education, Socioeconomic Status, and Social Capital (PROGRESS)-Plus determinant. We followed Preferred Reporting Items for Systematic Reviews and Meta-Analyses Extension for Scoping Reviews guidelines and registered the review with International Prospective Register of Systematic Reviews (CRD42021234567). Data were charted and analyzed thematically according to PROGRESS categories. A total of 163 studies were included. Most studies focused on education, socioeconomic status, and place of residence, whereas fewer addressed gender identity, sexual orientation, or disability. Common dietary indicators included fruit and vegetable intake, dietary patterns, and food group consumption. Evidence consistently showed that lower education and income levels were associated with poorer dietary outcomes. Notably, certain population groups (for example, ethnic minorities, rural residents, and individuals with low education or income) experienced cumulative disadvantages. The scoping review highlights persistent and intersecting diet-related health inequalities in high-income countries. It underscores the need for standardized indicators and intersectional approaches in monitoring, research, and policy making.

**Statement of significance**

This scoping review offers the first comprehensive synthesis of observational evidence on diet-related health inequalities using the PROGRESS-Plus framework. It identifies major evidence gaps and calls for stronger, equity-focused dietary monitoring systems in high-income countries.

## Introduction

Diet is among the major determinants of health. In 2017, dietary risks were responsible for 11 million deaths and 255 million disability-adjusted life years (DALYs), thereby making them responsible for more deaths than any other risk globally, including tobacco smoking [1]. Cardiovascular disease (CVD) was the leading cause of diet-related deaths and DALYs, followed by cancers and type 2 diabetes. However, neither the specific risks nor their impact are equally distributed [2,3]. Across the globe, variations in how populations eat exist based on aspects such as culture, climate, food production, and overall sociodemographic and economic aspects [4].

What characterizes diet-related health inequalities in front of other types of differences is that they arise from the social conditions in which people are born, grow, live, work and age, and are, by definition, unjust, unfair, and avoidable [5]. Diet-related inequalities can be defined as differences in dietary intake, dietary behaviors, and dietary patterns in different segments of the population resulting in poorer dietary quality and inferior health outcomes for certain groups and an unequal burden in terms of disease incidence, morbidity, mortality, survival, and quality of life [6].

Inequalities in diet-related diseases like non-communicable diseases (NCD) are well established [[7], [8], [9]]. Population groups in the lowest socioeconomic positions (SEPs) have the highest prevalence of excess weight [[10], [11], [12]] cardiometabolic disorders [13], and are at an increased risk of certain types of cancer, especially preventable ones such as lung or cervical [14]. Different ethnic and racial groups exhibit varying prevalences of NCDs, such as type 2 diabetes [15], hypertension [16], and cancer [3], due to gene–environment interactions, which are further shaped by health behavior, socioeconomic factors, and disparities in healthcare access and treatment.

Dietary patterns have been identified to contribute 17%–21% of the SEP gradient in all-cause mortality, 7%–24% in cardiovascular disorders, and around 10% in metabolic disorders [13]. Indeed, socioeconomic groups display intake differences in vitamins C and D, carotenes, calcium, and fiber intake [17,18], ultraprocessed food (UPF) consumption [19,20] and overall diet quality [21]. Socioeconomic dietary inequalities start before 24 mo of age [22], contributing to the unequal burden of childhood obesity-related conditions like hypertension, metabolic syndrome, and nonalcoholic fatty liver disease [23].

Factors that explain those differences span all the range of food choice determinants [24]. At the individual-level, they include exposure to foods with different sensory and perceptual features, as well as personal factors such as psychological states—like knowledge, attitudes, anticipated consequences, and habits [[25], [26], [27]]. Over the lifespan, these factors are shaped at a population level by broader cultural, economic, political and commercial forces, which lead to unequal exposures to both health promoting and health-damaging dietary behaviors by influencing key determinants such as cost [[28], [29], [30], [31], [32]], availability [[33], [34], [35]], convenience [36], material and informational/educational resources [[37], [38], [39]], marketing exposure [36,40], and symbolic representations of food [27,[41], [42], [43]], among others.

In other words, living conditions can generate a social gradient in diet quality that contributes to health inequalities [44]. Therefore, improving knowledge of the relationship between them appears to be a particularly relevant focus for health policy aimed at reducing unfair differences in diet and related health inequalities [27,[45], [46], [47]].

Several reviews have been published on the topic. However, they either focus exclusively on socioeconomic conditions [[48], [49], [50], [51], [52], [53], [54], [55], [56], [57], [58]], address diet-related inequalities from a global perspective [59] or have been published more than a decade ago [6,60,61]. Our study aims to fill existing gaps in the literature by offering a contemporary analysis of diet-related health inequalities within the context of high-income countries [62] and across the lifespan, underpinned by a comprehensive and scoping approach facilitated by the Place of Residence, Race/Ethnicity, Occupation, Gender/Sex, Religion, Education, Socioeconomic Status, and Social Capital (PROGRESS) framework [63]. Specifically, our review seeks to identify the diet-related health inequalities reported in recent literature, whereas also exploring the mechanisms driving these inequalities and the strategies proposed to address them.

By focusing on high-income countries, we recognize the varied contextual and upstream factors influencing diet and inequalities [64], and the impact of nutrition transition [65]. In turn, using the PROGRESS framework ensures a comprehensive examination of key characteristics shaping opportunities and health outcomes, including place of residence, race/ethnicity, occupation, gender/sex, religion, education, socioeconomic status (SES), and social capital. Through this approach, we aim to contribute to a deeper understanding of the complex relationship between diet, sociodemographic factors, and health inequalities, focusing on the prevalence rates and effect sizes based on the Social Determinants of Health (SDoH), underlying mechanisms, and potential effective strategies to address diet-related health inequalities.

## Methods

This article reports a scoping review on diet-related health inequalities in high-income countries following the methodological framework described by Arksey and O’Malley. We also followed the Guidelines published by the Joanna Briggs Institute as well as the PRISMA-ScR [[66], [67], [68]]. Along the process, the PROGRESS framework [63] is used to systematically and comprehensively identify social determinants of interest, as previously done by other researchers [69], and also use it to analyze and present the results related to the association between the different SDoH and diet intake indicators. This review has been registered in PROSPERO (CRD42023427685).

### Search strategy

The search strategy departed from the population, exposure, comparator, outcome research question, defined as: P: among people living in high-income countries, what is the effect of; E: being disadvantaged based on SDoH (based on the PROGRESS Framework) compared with; C: not being disadvantaged on O: dietary inequalities measured as food consumption. We did not apply a systematic approach to define “disadvantaged” for each characteristic, but rather relied on what the individual studies included in our systematic review identified as populations more prone to diet-related inequalities. The detailed search strategy was developed with the help of a medical librarian and is outlined in the online Supplemental File 1.

We searched PubMed, Scopus, and Web of Science for relevant observational studies published 2009 through 2023. The search was run again in June 2024 without additional results that fulfilled the eligibility criteria.

### Eligibility criteria

We included original observational studies conducted in high-income countries [62] from 2008 onwards analyzing differences in dietary intake based on the SDoH, as described in the PROGRESS Framework. Dietary intake had to be reported in terms of dietary patterns, food consumption, meal consumption or nutrient intake. Articles concerning diseases or groups of people with baseline disabilities, or studies on food insecurity either as an exposure or outcome were not included. We included articles written in the languages spoken by the research team: English, Spanish, Portuguese, French, and Polish.

Using Covidence software (Veritas Health Innovation, 2023), all publications were first title and abstract, and then full-text reviewed by pairs of the research team. Discrepancies were resolved by consensus of all the authors.

### Data extraction

The same investigators independently abstracted the articles that met the selection criteria, resolving any discrepancies by consensus. The authors of the original studies were contacted if relevant information on eligibility or key study data was not available in the published report.

The following information was recorded from all studies: country, study design, sample characteristics (size, age, gender, data source), objective, exposure variables (such as place of residence, race, occupation, gender, religion, education, SES, and social capital), covariates, outcome variables (patterns, food consumption, nutrient consumption,), size effect, results, mechanisms that explain the disparities (if described in the original article), possible strategies that are discussed in the article (if described in the original article).

Mixed Methods Appraisal Tool (MMAT) [70] was used to assess the quality of the included observational studies.

### Data analysis

Although we attempted to conduct a meta-analysis, it was not feasible because of data limitations and study heterogeneity. Therefore, data analysis was conducted in the form of a narrative synthesis structured based on the PROGRESS framework categories. Study categorization followed the definitions of each dimension proposed by the Cochrane Equity Studies [63] using occupation to derive social position were categorized within the SES PROGRESS category, observing the evidence that shows that occupation and social class are 2 distinct constructs that lead to different hypotheses of social stratification, social mechanisms, and intervention strategies regarding health inequalities, and they should, therefore, be studied separately [71]

## Results

### Descriptive results

#### Study selection

**FIGURE 1:**
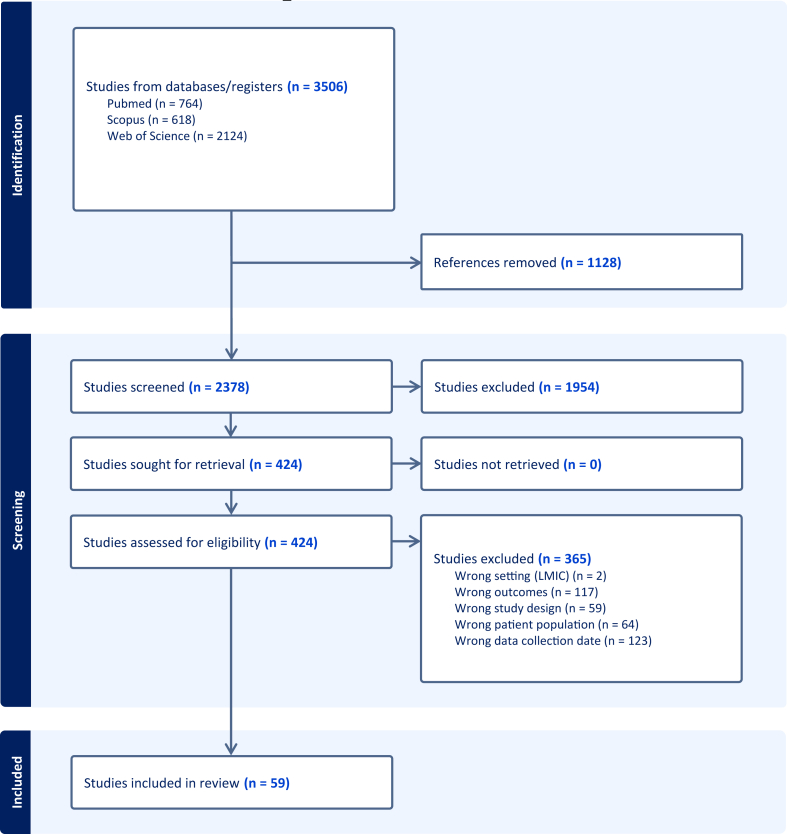
PRISMA flowchart showing the number of publications identified, screened, assessed for eligibility, and included in the review, with reasons for exclusion at each stage. LMIC, low- and middle-income countries.

Figure 1 shows the number of publications identified, screened, assessed for eligibility and included, with reasons for exclusion at each stage. A total of 3506 publications were initially identified. After removing duplicates (*n* = 1128), 2378 references were title/abstract screened, of which 1954 leaving 424 studies for full-text eligibility. Of those, 365 studies were excluded because data collection had taken place before 2009 (*n* = 123), reported nondietary outcomes (*n* = 117), were not observational or did not report original data (*n* = 59), were conducted in clinical populations, or did not have a reference group to compare (*n* = 64), or reported data from low- and middle-income countries (*n* = 2). Additionally, 7 references were excluded because full-text was not available. The final sample comprised 59 publications.

The complete extracted information from these 59 publications is in the Supplemental Table 1.

#### Study characteristics

All eligible publications described observational data. More than half of the sample (*n* = 34) reported data from the United States. The rest of the studies were conducted in Australia (*n* = 6), United Kingdom (*n* = 3), The Netherlands (*n* = 2), Spain (*n* = 2), Japan (*n* = 2), and Belgium, Denmark, France, Ireland, Israel, Italy, Korea, and Norway, each with 1 study. Two articles reported data from various countries [72,73].

Most of the samples were cross-sectional studies (45 of 58), and all of them used individual-level dietary data. Most exposures were also collected at this level, with a few articles including contextual data (that is, area poverty level, food availability).

Twenty-one articles reported data on adults [3 exclusively in male population and 6 in females, including pregnancy and postpartum (*n* = 3)], 17 on children, 5 on children and adolescents, 8 on adolescents, and 1 in elderly. Cut-offs for age categories were made based on the WHO definitions of adolescent (10–19 y old) and elderly (older than 60 y old) [74,75]. Two studies examined household dietary intake, and 1 in young adults, including university students. An article reported whole population data [76]. Sample size ranged from 131 [44] to 700,000 (data from 34 countries) [72]. Most studies’ samples ranged between 1000 and 10,000 participants.

Eligible studies focused on dietary patterns (*n* = 22), measured through different quality diet index; consumption of certain food groups or products (*n* = 33), or nutrient intake (*n* = 11). Four assessed breakfast consumption and 3 others analyzed breastfeeding; consumption of an evening meal, eating at a restaurant on the previous day; and family dinners, respectively. Sixteen studies combined several dietary outcomes.

In terms of exposures, 19 studies analyzed aspects of SES, including education, occupation, income, and contextual conditions, alone or in combination. Ethnicity was the most investigated exposure, with 23 studies, alone (*n* = 11) or combined with indicators of SES (*n* = 12). Gender and sexual orientation were the exposure in 2 articles, and residential characteristics in other 8. Seven combined multiple exposures across dimensions. Only 1 investigated religion, together with ethnicity.

The quality of the studies included was analyzed following the criteria of the MMAT tool (Supplemental Table 2). In general, studies did fulfill the quality criteria described in the MMAT instrument, although in some cases data on sample representativeness, risk of non-response bias or sampling strategy was missing. Following the MMAT guidelines, we did not exclude studies based on methodological quality alone, as this is generally discouraged. Instead, we documented the quality assessments in detail and considered them in our narrative synthesis. This approach ensures transparency and allows for a more comprehensive understanding of the evidence, and accounting for potential methodological limitations.

### Results by exposure

#### Place of residence (P)

##### Area level deprivation

The effect of area deprivation on diet inequalities was investigated by 4 papers across various age groups in Australia [77,78], children in the United States [79], and adult females in Ireland [80].

Among adolescents, Niven et al. [77] found that those living in lower-SEP areas had higher odds of poor dietary habits, including low vegetable intake and frequent consumption of sugar-sweetened beverages (SSB) and fast food [odd ratio (OR) 1.29–1.48]. Additionally, Wilson et al. [78], using data from the Childhood Determinants of Adult Health, found that socioeconomic mobility influenced adult diet quality. Individuals with stable low education or downward mobility had significantly lower Dietary Guidelines Index (DGI) scores—5.5 points lower in males and 6.3 in females. Eagle et al. [79] observed that as area household income decreased, fried food consumption doubled (from 0.23 to 0.54 times/d, *P* < 0.002), whereas vegetable intake declined. Similarly, McCartney et al. [80] reported that young Irish females from low-SES backgrounds had less favorable diets, including lower intake of fruits, vegetables, and whole grains, and higher consumption of processed meats, SSBs, and fried foods.

These patterns were shaped by multiple factors, including limited access to healthy food, greater exposure to fast-food outlets, poor urban infrastructure, low perceived control, and weaker health-related attitudes. Eagle et al. [79] highlighted the influence of educational attainment and family structure, whereas Wilson emphasized peer effects and income-related differences in health literacy and food access. Livingstone et al. [81] also suggested that gender disparities in diet quality scores may explain inconsistent associations between income and dietary habits.

To address these challenges, the authors suggest interventions should focus on improving access to education, implementing targeted health education campaigns, and implementing government policies to alleviate financial barriers to healthy eating.

##### Rural/urban

Three studies from Australia [82], the United States [83], and Korea [84] explored disparities in diet quality and food consumption among females of reproductive age, adults, and elderly populations, respectively, across urban and rural settings.

In Australia, Martin et al. [82] found no significant differences in diet quality between urban and rural females [adjusted *β* = –1.8; 95% confidence interval (CI): –5.1, 1.4]. However, higher income (≥$AUD80,000: *β* = 5.5; 95% CI: 1.2, 9.8) and employment status (unemployed: *β* = –4.1; 95% CI: –8.1, –0.14) were positively associated with better dietary outcomes, regardless of residence. Conversely, Lutfiyya et al. [83] reported that rural United States adults were significantly less likely to consume ≥5 servings of fruits and vegetables daily compared with nonrural adults (OR = 1.161; 95% CI: 1.160, 1.162), with <25% meeting this recommendation. Within rural populations, higher intake was associated with being female, non-Caucasian, partnered, more educated, physically active, and maintaining a healthy BMI.

In Korea, Park et al. [84] observed lower diet quality scores in rural areas compared with urban areas (total rural compared with urban: *β* –2.6, SE 0.6, *P* < 0.001). Determinants of diet quality varied by setting: in urban areas, higher income (*β* = 0.9; *P* = 0.007) and home ownership (*β* = 3.6; *P* < 0.001) were positively associated with diet quality, whereas in rural areas, food insecurity (*β* = –5.8; *P* < 0.001) and older age (*β* = –0.4; *P* < 0.001) were linked to poorer dietary outcomes.

Across studies, key mechanisms included SES, employment, access to healthy food options, and broader environmental constraints. Suggested strategies included healthy food vouchers and supplemental food packages [82], improving access to healthy foods in rural areas [83], and tailoring interventions to individual characteristics and local food environments [84].

#### Race/ethnicity (R)

##### Race/ethnicity

Eleven studies, all conducted in the United States, examined dietary inequalities based on race/ethnicity. Six of them focused on children, 4 on adolescents, and 1 on adults.

Guerrero and Chung [85] found significant dietary differences among California children aged 2–11 y across racial/ethnic groups, with Asian children being more likely than Whites to have low fruit and vegetable intake, and Latino children more likely to consume more fruit juice but fewer sweets [85]. Welker et al. [86] also reported significant differences in the consumption of dairy products, meat products, and SSB across ethnics of 2- and 3-y olds [86]. Highland et al. [87] examined ethnic differences in parental health beliefs and their relation to children’s health behaviors among Latina and non-Latina mothers with children aged under 18 y [87]. Children of Latina mothers consumed more soda and fried foods and were less physically active. Latina mothers were also more likely to perceive barriers to healthy eating and less likely to see its benefits. Maternal beliefs about healthy eating mediated the link between Latino ethnicity and higher soda consumption. Mendez et al. [88] used longitudinal data to examine disparities in SSB consumption among children and adolescents. Although overall intake declined from 2003–2004 to 2013–2014, racial/ethnic and income disparities persisted. Among non-Hispanic White children, higher income was linked to lower SSB intake. In contrast, non-Hispanic Black and Mexican-American children from high-income households consumed as much or more SSBs than their low-income peers. Fruit drink consumption was especially high among non-Hispanic Black children [88].

Haughton et al. [89] assessed racial/ethnic disparities in meeting daily nutrition and activity targets among children and adolescents. None of the adolescents and <1% of children met all 4 recommended targets. Significant differences were observed across racial/ethnic groups for specific behaviors. Asian adolescents, for example, were less likely than non-Hispanic Whites to meet fruit, vegetable, and physical activity targets, but more likely to meet the zero-SSB consumption target [89]. Ranjit et al. [90] investigated racial and ethnic differences in the home food environment and healthy eating among children and adolescents [90]. White children had significantly better food environments—greater availability of healthy foods, more family meals, and stronger parental support—than Hispanic and Black children. These differences were linked to healthier food consumption, though disparities in unhealthy food intake remained after adjusting for home environment. The authors suggested unmeasured cultural and socioeconomic factors—such as time constraints, stress, and culturally shaped food practices—as possible explanations.

Also among adolescents, Bekelman et al. [91] compared diet quality and snack intakes between non-White Hispanic and Mexican-American adolescents of varying acculturation levels [91]. They found that non-Hispanic White adolescents consumed larger snack portions and obtained more daily energy from snacks than most Mexican-American acculturation groups. Larson et al. [92] explored ethnic/racial differences in the home/family environments of adolescents and their association with dietary intake and weight status [92]. Parental encouragement for healthy eating was found to be associated with SSB intake among youth representing certain ethnic/racial groups. Among adults, Hauschildt and Burgard [93] found that racial differences in dietary behaviors among older United States adults were partly explained by levels of social integration—including communication, social support, employment, and community involvement [93]. Additionally, Poti et al. [94] reported that Black households purchased significantly fewer highly processed and ready-to-eat foods than White households.

In a study exclusively focusing on males, Thompson et al. [95] compared diet quality between non-Hispanic Black and White males. After adjusting for sociodemographic factors, overall diet quality was similar; however, Black males had lower scores for vegetables, seafood/plant proteins, and dairy, but higher scores for fruit, total protein, and fatty acids [92].

##### Race/ethnicity combined

Twelve studies analyzed the interrelation of ethnicity combined with other SES factors in their association with dietary intake. Four refer to adults, 2 to pregnant females, 3 to children, 2 to adolescents, 1 to the whole population, and 1 to households. All studies but 2 [96,97] were conducted in the United States.

The association of ethnicity and educational attainment on dietary intake was analyzed by 3 studies. Rashid et al. [96] used Dutch cohort data to examine dietary patterns in 5-y olds. They identified 4 distinct patterns, with non-Dutch children more likely to follow “snacking” and “healthy” patterns. Turkish children scored higher on full-fat, and Surinamese on meat-heavy diets. Children of less-educated mothers were more likely to follow snacking and meat patterns. In the United States, Assari et al. [98] found that higher maternal education was linked to more frequent breakfast consumption among White youth, but not among Black youth—suggesting that the protective effect of education differed by race [98]. Lee and Seon [99] found that education had opposite associations with fast food intake across ethnic groups in United States males. Among African Americans, higher education predicted greater fast food consumption, whereas among non-Hispanic Whites and Hispanics, it predicted lower consumption [99].

Cooksey Stowers et al. [100] and Newby et al. [101] explored geographical inequalities and race/ethnicity variations. Cooksey Stowers et al. [100] focused on perceptions of residing in food deserts and food swamps among lower-income and racial/ethnic-minority individuals. Residents of food deserts (OR = 0.74, *P* < 0.05) and food swamps (OR = 0.75, *P* < 0.001) had lower diet quality scores. Among non-Hispanic Blacks, those in food swamp areas (OR = 0.66, *P* < 0.01) reported lower diet quality. No significant differences were found in the Hispanic subsample. In a completely male sample, Newby et al. [101] found significant differences in nutrient intake patterns between Black and White males living in the Stroke Belt (region in the southeastern states where stroke incidence and mortality rates are significantly higher than the national average) compared with other United States regions [101]. Black males consumed less trans-fat than White males only in that region (*β* = –0.21; 95% CI: –0.31, –0.11). They also had lower intakes of sodium, potassium, magnesium, and calcium, but higher cholesterol intake (all *P* < 0.05). Males in the Stroke Buckle had the lowest intakes of fiber, potassium, magnesium, and calcium, whereas those in both the Stroke Buckle and Stroke Belt had higher cholesterol intake compared with other regions (*P* < 0.005).

In a population cohort, Liu et al. [76] found disparities in junk food consumption trends across demographic factors like sex, race/ethnicity, education, and income, particularly highlighting variations in food obtained from grocery stores. Raffensperger et al. [102] concluded that education, income, and employment were significant predictors of nutrient-based diet quality [assessed by mean adequacy ratio (MAR), a measure of nutrient-based diet quality that evaluates the degree in which multiple nutrients meet recommended intakes] for African Americans, whereas sex, education, and smoking status were significant predictors for Whites [102].

Wang et al. [103] explored fruit and vegetable intake among United States females. Race/ethnicity, education, and income were independently associated with lower intake, but neighborhood poverty was not. Females of color, high school graduates, and those with incomes at 301%–400% of the federal poverty level were more likely to consume fruits and vegetables less than once a week than White, college-educated females with higher incomes. Patterns were similar among immigrant and United States–born Latinas [103]. Brunst et al. [104] identified ethnicity, education, and income as significant determinants of micronutrient adequacy [104]. Factors associated with multiple antioxidant inadequacies included being Hispanic or African American, lower education, and self-reported economic related food insecurity. Hispanics had a higher prevalence of multiple methyl-nutrient inadequacies compared with African Americans; both had suboptimal betaine intakes and higher odds for vitamin B_6_ and Fe inadequacies compared with Caucasians.

Woolf et al. [105] found that non-White youth were significantly less likely to consume healthy proteins compared with White youth (OR = 0.71, 95% CI: 0.55, 0.92) and significantly more likely to eat at a restaurant (OR = 1.32, 95% CI: 1.02, 1.70) [105]. Lower SES youth were significantly less likely to eat an evening meal compared with higher-income youth (OR = 0.59, 95% CI: 0.39, 0.89). In Belgian adolescents, Rouche et al. [97] found differences in the consumption of several food groups among first and second generation status. Moreover, adolescents with a low family affluence scale (FAS) were more likely to consume chips and fries at least once a day compared with those with a high FAS, with natives showing an adjusted relative risk ratio of 1.39 (95% CI: 1.12, 1.73).

Proposed mechanisms explaining racial/ethnic inequalities on dietary intake include acculturation status and cultural factors influencing dietary practices, such as the role of cultural heritage and family environment [85,91,97]. Socioeconomic factors, including education level, poverty, and household structure, also play significant roles [93,95,100,101]. Other contributors include access to healthy foods, cost, marketing exposure, and limited physical availability [94,105]. Beliefs about health, perceived risks, and nutrition knowledge further influence food choices [93,106]. Structural barriers—such as limited upward mobility, food insecurity, and differences in time, skills, and food access across cultural and religious groups—compound these challenges [88,91,97,98,106]. Addressing these multifaceted factors requires comprehensive and culturally tailored interventions aimed at promoting equitable access to healthy foods and addressing socioenvironmental determinants of dietary behaviors.

A wide range of strategies has been proposed to reduce racial and ethnic disparities in dietary intake, spanning individual, community, and policy levels. At the individual and family level, recommendations include promoting healthier snacking habits among adolescents to reduce acculturation-related risks [91], supporting Latino youth in switching from soda to water [87], and offering culturally tailored dietary counseling and prenatal nutrition education [85,102,104]. Creating supportive home environments and encouraging parents from diverse backgrounds to follow dietary recommendations are also key [92]. Community-level strategies involve improving access to affordable physical activity facilities and delivering customized nutrition education [107], as well as integrating cultural and social factors—such as informal integration—into interventions [93]. At the structural level, proposed actions include addressing disparities in access to healthy food outlets through targeted policy efforts [100], advancing nutrition equity via education and anticipatory guidance [99], and designing interventions that both improve nutrient adequacy and reduce excesses in specific populations [106]. Emphasis has also been placed on addressing broader issues like minority/marginalized group discrimination and barriers to upward mobility [98].

#### Occupation (O)

No study analyzed the relationship between occupation and dietary intake as the main aim. Studies including occupation as an indicator for SES are included in the corresponding section.

#### Gender/sex (G)

Two studies conducted in the United States aimed to explore disparities in eating behaviors and diet quality among adolescents and young adults based on sexual orientation and gender expression [108,109].

Luk et al. [108] identified significant differences in diet quality by gender expression and sexual orientation. Sexual minority males consumed fruits and vegetables 1.7 times more per week than heterosexual males, though intake of snacks, sodas, and whole grains was similar. In contrast, sexual minority females were more likely to be overweight, pointing to disparities in weight status by sexual orientation. It was found that gender-nonconforming males had higher diet quality scores than very gender-conforming males (*β* = 2.0, *P* < 0.05). Similarly, gay males and mostly heterosexual females had better diet scores than their exclusively heterosexual peers. However, gender-nonconforming females were less likely to eat breakfast than very gender-conforming females (*β* = 0.9, *P* < 0.05).

One explanation, according to Luk et al. [108], is the role of gender norms—particularly the perception of healthy eating as a “feminine” behavior. These norms may discourage some males from adopting healthy diets. Suggested strategies include framing nutrition messages in ways that challenge stereotypes, particularly those linking masculinity to unhealthy food choices.

#### Religion (R)

In our sample, only 1 study [107] address religion as a possible axis of dietary inequalities, reporting data from Israel. In the multivariate analysis, Arabs showed lower odds than Jews of being in the highest intake tertile for the healthy pattern (OR = 0.38, 95% CI: 0.18, 0.83), although this difference was less pronounced among diabetics. Higher education, physical activity, and past smoking were linked to healthier dietary patterns. The observed dietary differences reflected broader cultural and socioeconomic disparities between Arabs and Jews. However, younger and male Arab participants showed more similar dietary patterns to Jewish participants, suggesting the influence of acculturation. The authors recommended tailored interventions—such as specialized facilities, workplace programs, printed materials, and targeted social marketing—to address these inequalities.

#### Education (E)

Four studies investigated the association between educational attainment and dietary intake. Two of them [110,111] observed intergenerational relationships, a third focused on postpartum females [112] and the fourth one was conducted on general adult population [113]. Studies analyzing the interaction of education and other aspects of SES have been included in the (S) dimension.

Friis et al. [113] examined the role of health literacy in the link between education and diet among Danish adults. Individuals with lower educational attainment were 2.37 times more likely to follow an unhealthy dietary pattern (95% CI: 2.12, 2.65). Health literacy—especially the ability to understand health information—partially mediated this association, explaining 13% of the variance. Additional contributors included social norms, workplace environments, stressors, and income. In postpartum females, Martin et al. [82] found that greater acculturation was associated with lower fruit and vegetable intake, poorer Healthy Eating Index scores, and higher postpartum weight retention—averaging 0.8 kg gained per 1-unit increase in acculturation.

Van Ansem et al. [111] found that in The Netherlands children of mothers with higher education levels consumed more fruits and vegetables, with an effect size of 0.13 (95% CI: 0.04, 0.22) for fruit consumption and 23.81 g (95% CI: 14.93, 32.69) for vegetable consumption. Additionally, these children were nearly 3 times more likely to have breakfast daily (OR = 2.97, 95% CI: 1.38, 6.39) compared with children of less-educated mothers. In young United States females, Lee et al. [110] observed that higher maternal education was associated with reduced fast food intake among daughters, with coefficients of 0.16 (SE = 0.08) for mothers and -0.23 (SE = 0.09) for young females.

The studies reviewed highlight several mechanisms underlying dietary disparities, including low health literacy [113], acculturation [82], and household food environments shaped by parental education [111]. Lee et al. [110] further emphasized the intergenerational effects of maternal education on young females' fast food intake. Additional influences included social norms, workplace conditions, and economic stressors [113]. Proposed strategies include interventions targeting parental nutritional knowledge and health literacy among adults and policy measures aimed at improving educational attainment, particularly among parents.

#### Socioeconomic status (S)

##### Socioeconomic position

Two studies conducted delved into the intersection of SEP and dietary behaviors among adolescents. Czhen’s cohort study, spanning 34 countries, uses the FAS to elucidate SEP influences in diet [72]. In Spain, Esquius et al. [114] found a higher prevalence of breakfast skipping among adolescents from lower SEP backgrounds, especially girls, using a subjective SEP scale. A third study, by Livingstone et al. [81], delves on SEP in Australian adults [81], observing that individuals from lower SES backgrounds had significantly lower DGI scores—≤4.5 points lower by education level and 2.5 points lower by income—indicating poorer diet quality across education and income strata.

Variations in nutritional knowledge and unequal access to resources were identified as key factors contributing to these disparities globally. Proposed strategies include national and international policies to promote equitable access to resources and adapting public policies to consider socioeconomic perspectives, particularly gender, to mitigate nutritional and health inequalities among adolescents.

##### Income

Four studies delved into the relationship between socioeconomic factors and dietary habits across different age groups.

Using data from the UK Millennium Cohort Study, Noonan [115] found that adolescents living in poverty consumed more unhealthy foods—including sweetened drinks and fast food—and had nearly double the odds of low fruit intake (OR = 1.92; 95% CI: 1.73, 2.13). Murayama et al. [116] found similar disparities among Japanese schoolchildren, associating lower household income with reduced intake of protein-rich foods and green vegetables. Among those aged 15–18 y, mean dietary quality scores ranged from 44.3 (95% CI: 39.0, 49.7) in the lowest income group to 51.6 (95% CI: 49.7, 53.4) in the highest. Also, in Japanese children, Kurotani et al. [117] observed no diet–income association among children aged 6–14 y, likely due to universal school meals. However, among 15–18-y olds, lower-income youth consumed fewer vegetables and less fiber and potassium, with a marginal link to lower fruit intake.

In United States adults, Zagorsky and Smith [118] did not identify a clear socioeconomic gradient in fast-food consumption, based on income and wealth. Their results showed that fast-food consumption increased with income up to the middle quintile, but overall differences across income and wealth levels were small—wealthier adults consumed ∼1 fewer fast-food meal per week than those in the lowest quintile. Other factors, such as food label use and work hours, were likely more influential.

Economic constraints of poverty were identified as a primary factor contributing to poor dietary intake among adolescents [115]. To address this, authors called for policy interventions targeting poverty-linked dietary inequalities. Japanese findings highlighted the importance of subsidized school meals for lower-income youth, especially in high schools without lunch programs [81,114]. In adults, geographically targeted interventions were proposed to address dietary disparities [118].

##### Income and education

Six studies analyzed the interrelation of income and education in their association with dietary intake.

Among adults in Australia, Miura et al. [119] found no clear link between household income and takeaway food consumption. However, adults without postsecondary education were more likely to consume takeaway >4 times per month (PR = 1.26; 95% CI: 1.03, 1.54) compared with university graduates [119]. Aggarwal et al. [120] showed that higher income and education were associated with lower energy-dense diets and higher nutrient adequacy (MAR scores) in the United States, with diet cost but no education mediating these associations [120]. In Italy, Cavaliere et al. [121] identified both income and education to be associated with a higher adherence to Mediterranean diet, with education holding a greater influence [*β* = 0.26 (*P* = 0.001) compared with 2.869 (*P* = 0.001)] [121]. VanKim and Laska [109] found that United States participants whose parents lacked a high school diploma were less likely to consume fruits and vegetables (OR = 0.80; 95% CI: 0.76, 0.84), whereas financial strain did not significantly affect dietary outcomes [109].

In children, Manyanga et al. [73] found that in 7 of 12 countries, lower income was significantly associated with unhealthier dietary patterns in 9–11-y olds. Using longitudinal data from Norway, Bolt-Evensen et al. [122] found that participants with a higher educational level in adulthood and higher educational intentions in adolescence had a significantly lower frequency of consumption of SSB at all-time points [122]. No differences were found based on income, nor significant widening (or narrowing) of inequalities were observed from childhood to adulthood.

The mechanisms explaining the interrelation of income and education in their association with dietary intake involve a complex interplay of factors, including nutritional knowledge and socioeconomic disparities. Studies like Miura et al. [119] and Van Kim [109] suggest an inverse relationship between education and takeaway consumption, although inconsistencies arise due to varying definitions of takeaway foods, methods of measuring education, and specific dietary behaviors examined. Additionally, the affordability of unhealthy compared with healthy food choices significantly influences consumption patterns, with lower-income individuals often constrained to less healthy options, as noted by Manyanga et al. [73]. This socioeconomic disparity is further evidenced by the association between income inequality and the consumption of unhealthy foods.

Efforts to enhance diet quality should prioritize addressing unhealthy dietary patterns and promoting healthier eating, whereas also tackling income disparities. Despite the availability of inexpensive takeaway options, household income might not substantially impact consumption habits, highlighting the intricate interaction between nutritional knowledge, socioeconomic factors, and dietary behaviors. Proposed strategies include public health measures such as taxing sugary beverages, restricting food marketing to children, and enhancing school meal standards [122]. Promoting affordable, traditional diets and addressing food literacy and income inequality is essential. As Manyanga et al. [73] note, the global spread of UPF continues to drive income-based dietary gaps—even in less developed settings—highlighting the need for sustainable, equity-oriented dietary policies worldwide [120,123].

##### Education and occupation

Martínez-Martínez et al. [124] examined the relationship between parental education and occupation and children's fish intake. They found no significant association with the father's occupation, except for shellfish consumption (*P* = 0.01). In contrast, the mother's occupation was significantly associated with both lean fish (*P* = 0.01) and fatty fish intake (*P* = 0.04). Parental education levels showed no significant impact. The authors recommended dietary interventions focused on increasing fish and seafood consumption in children, particularly those that address gender-based differences in parental roles and influence.

##### Income, occupation and education

Two studies explored the effect of the 3 most used indicators of SES in relation to dietary intake, focusing on animal foods intake [125,126] and dietary approaches to stop hypertension (DASH) scores [126].

Méjean et al. [125] found significant differences in animal foods intake based on education levels, with lower-educated individuals demonstrating higher consumption of red meat, processed meat, and poultry (red meat: +9 to 12 g/d; processed meat: +6 to 9 g/d; poultry: +7 g/d in males). Patel et al. [126] reported overall improvements in DASH scores over time, but persistent socioeconomic gaps remained—particularly in the intake of fruits, vegetables, whole grains, nuts, seeds, and legumes. Although diet quality increased across all groups, differences by SEP persisted, limiting progress in reducing socioeconomic inequalities in noncommunicable diseases, especially CVD.

Education emerged as a strong predictor of dietary habits, shaping occupation, income, and related health behaviors. Cultural norms and financial constraints also influenced food choices. To address these disparities, targeted strategies are needed to improve nutrition knowledge and facilitate access to healthy foods—especially among disadvantaged populations. Recommended actions include education campaigns, policy measures to improve affordability, and initiatives to reduce cost-related barriers to healthy eating.

#### Social capital (S)

No studies explored the role of social capital on dietary inequalities.

##### Multiple

Across various age groups, several studies aim to elucidate the interplay between different sociodemographic factors and dietary behaviors. Among children aged 1–13 y, investigations by Moore et al. [127] and Zarnowiecki et al. [128] uncover disparities in nutrient intake and healthy eating patterns. Moore's study underscores income and racial differentials in vitamin D consumption, notably revealing higher intakes among affluent and NH White populations. Zarnowiecki et al.'s findings [128] reported that fruit and vegetable consumption was influenced by self-efficacy and household dynamics, with income moderating these associations—especially among boys.

In adolescents, Morgan et al. [129] and Mendez et al. [88] investigate consumption trends of sugar-SSBs and energy drinks. Morgan et al. [129] identified gender and socioeconomic differences in SSB intake, with girls and adolescents from lower SEP consuming fewer SSBs. Mendez et al. [88] documented an overall decline in SSB consumption but noted persistent disparities by income and race/ethnicity. Notably, non-Hispanic White children from low-income households had higher SSB intake than their non-Hispanic Black peers.

In the area of maternal health, Parker et al. [84,106] analyzed data from the Infant Feeding Practices Study II and found that high-income females had higher prenatal diet quality (Alternative Healthy Eating Index for Pregnancy scores), particularly in moderation components (refined grains, sodium, and empty calories). Non-Hispanic Black females had lower overall scores than other groups.

Economic constraints may limit access to fortified foods, exacerbating differences in nutrient intake [127]. Tailored health promotion policies are needed to address the moderating effects of SEP on dietary behaviors [128]. Shifts in marketing trends and the availability of sugary beverages worsen consumption inequalities [126], whereas neighborhood environments significantly influence dietary habits, necessitating community-level interventions [130]. Racial/ethnic gaps in SSB consumption and prenatal diet quality further underscore the need for targeted strategies to reduce inequalities based on income and race/ethnicity [85,127].

Table 1 [72,73,[76], [77], [78], [79], [80], [81], [82], [83], [84], [85], [86], [87], [88], [89], [90], [91], [92], [93], [94], [95], [96], [97], [98], [99], [100], [101], [102], [103], [104], [105], [106], [107], [108], [109], [110], [111],[113], [114], [115], [116], [117], [118], [119], [120], [121], [122], [123], [124], [125], [126], [127], [128], [129],^131^] presents the main results of the sampled studies according to the PROGRESS dimensions and dietary intake outcomes. Table 2 summarizes the proposed mechanisms explaining diet-related health inequalities and the strategies suggested to mitigate them.

**TABLE 1:** Description of main results of the sampled studies, according to PROGRESS dimension and dietary intake outcome

		Food pattern	Food consumption	Nutrient consumption	Other
Place of residence	Area level deprivation	• Areas with low education and downward mobility during childhood and adolescence associated with notably lower Dietary Guidelines Index scores in Australian adults [78].	• Lower-SEP areas have higher odds ratios for high consumption of SSB and fast food, and low intake of vegetables [77]. • Areas with decreased household income in the United States display an increase in the frequency of fried food consumption and a decreased vegetable consumption [79]. • Young Irish females with low-SES show less favorable dietary patterns, including lower intake of fruits, vegetables, and whole grains, and higher intake of processed meats, SSB, and fried foods [80].	—	—
	Rural/urban	• In Australia, Martin et al. [82] stated no significant difference in diet quality between urban and rural females, which was instead associated with higher income and employment in both settings. • In Korea, Park et al. [84] observed lower diet quality scores in rural areas compared with urban areas. In both urban and rural areas, gender, education level, and socioeconomic factors significantly impact diet quality.	• United States [83] found rural adults are less likely to consume the recommended servings of fruits and vegetables compared with nonrural adults, with <25% of rural adults meeting the guideline. Among rural adults, increased fruit and vegetable consumption was associated with being female, non-Caucasian, married or partnered, having higher income or educational attainment	—	—
Race	Ethnicity (adjusted)	• Bekelman et al. [91] found that non-Hispanic White adolescents had modestly poorer diet quality as measured by HEI-2015 compared with United States-born adolescents with a foreign-born parent and Mexican-born adolescents. • Thompson et al. [95] found that although overall diet quality did not differ significantly after adjusting for sociodemographic measures, non-Hispanic Black males had lower scores for vegetables, seafood and plant protein, and dairy, but higher scores for fruit, total protein, and fatty acids compared with non-Hispanic White males.	• Guerrero et al. [85] found significant differences among California children, with Asians having lower vegetable and fruit intake, and Latinos consuming more fruit juice but fewer sweets than Whites. • Welker et al. [86] reported disparities in dairy, meat, and sugar-sweetened beverage consumption among 2- and 3-y olds. • Haughton et al. [89] noted that Asian adolescents had lower rates of meeting physical activity and fruit and vegetable consumption targets but were more likely to meet the zero sugar-sweetened beverage target compared with non-Hispanic Whites. • Highland et al. [87] noted that children of Latina mothers consumed more soda and fried foods and exercised less than those of non-Latina mothers. • Mendez et al. [88] highlighted persistent disparities in sugar-sweetened beverage intake, with higher consumption among non-Hispanic Black and Mexican-American children from affluent households. • Ranjit et al. [90] found that White children had significantly better home food environments compared with Hispanic and Black children, with disparities in healthy food consumption but not in unhealthy food consumption. • Larson et al. [92] linked parental encouragement for healthy eating to lower sugar-sweetened beverage intake among adolescents from certain ethnic/racial groups. • Poti et al. [94] found Black households purchased fewer highly processed foods compared with White households. • Hauschildt et al. [93] showed racial differences in fruit and vegetable consumption among older adults influenced by social integration. • Bekelman et al. [91] found that Non-Hispanic White adolescents had higher total snack intake than most language use groups, except those with equal Spanish and English use at home.	—	—
	Ethnicity (combined)	• Rashid et al. [96] identified dietary patterns in Dutch children, linking them to ethnicity and education. Non-Dutch children preferred snacking and healthy foods, whereas Turkish and Surinamese children favored full-fat and meat patterns. Children of less-educated mothers scored higher on snacking and meat patterns. • Stowers et al. [100] found that lower-income and minority individuals perceived food deserts and swamps, correlating with lower diet quality. • Liu et al. [76] found that, between 2003 and 2018, the proportion of individuals consuming poor-quality diets from grocery stores decreased among non-Hispanic White and non-Hispanic Black adults, whereas it remained stable for Hispanic adults. • Raffensperger et al. [102] found that diet quality in African Americans was predicted by education, income, and employment, whereas in Whites, it was predicted by sex, education, and smoking status.	• Lee et al. [99] observed that higher-educated African-American males in the United States consumed more fast food, whereas higher-educated non-Hispanic Whites and Hispanics consumed less. • Rouche et al. [97] found that Belgian adolescents with low family affluence, especially first and second-generation individuals, consumed chips and fries daily. • Wang et al. [103] found that females of color and those with lower education and income were more likely to consume fewer fruits and vegetables, with race/ethnicity, education, and income being key factors. • Woolf et al. [105] reported that minority youth ate at restaurants more often and consumed fewer healthy proteins than White youth.	• Newby et al. [101] noted regional nutrient intake differences between Black and White males in the United States, with disparities in trans-fat, sodium, and other nutrients. • Raffensperger et al. [102] found that African Americans had lower micronutrient adequacy. • Brunst et al. [104] highlighted that ethnicity, education, and income determined micronutrient adequacy in pregnant females, with Hispanics and African Americans showing higher nutrient inadequacies compared with Caucasians.	• Assari et al. [98] found a positive association between maternal educational attainment and breakfast frequency among White youth, whereas no significant correlation was observed among Black youth, suggesting differentiated impacts of maternal education on breakfast frequency within Black families. • Woolf et al. [105] found that socioeconomic status youth were less likely to have evening meals.
Occupation		Studies including occupation as an indicator for socioeconomic status are included in the corresponding section.			
Gender/sex		• In Van Kim et al. [109] study, gender-nonconforming males had notably higher diet quality scores than very gender-conforming males, suggesting a more nutritious overall diet among those who do not conform to traditional gender norms	• According to Luk et al. [108], sexual minority males consumed fruits and vegetables more frequently than their heterosexual peers but showed similar intake levels of snacks, sodas, and whole grains. Conversely, sexual minority females were more likely to be overweight.	—	—
Religion		• The study by Bayram and Donchin [107] found that Arabs showed lower odds than Jews of being in the highest intake tertile for the healthy pattern.		—	—
Education		• Friis et al. [113] found that lower educational attainment was associated with a 2.37 times higher likelihood of adhering to an unhealthy dietary pattern in Danish adults.	• Martin et al. [82] observed that higher levels of acculturation were associated with lower intakes of fruits and vegetables in postpartum females. • Van Ansem [111] reported that children of mothers with higher education levels consumed significantly more fruits and vegetables in The Netherlands. • Lee [110] showed that higher maternal education was linked to reduced fast food intake among young United States females.	—	• Van Ansem [111] reported that children of mothers with higher education levels were more likely to have breakfast daily
Socioeconomic status	Socioeconomic position	• Livingstone et al. [81] found that Australian adults in disadvantaged areas with lower socioeconomic status had significantly lower diet quality, with Dietary Guidelines Index scores ≤4.5 units lower for education and 2.5 units lower for income.	• Chzhen et al.’s [72] found that adolescents from less affluent families were more likely to exhibit poor dietary behaviors, such as consuming fewer fruits and vegetables.	—	• In Spain Esquius, [114], a higher prevalence of skipping breakfast was found among adolescents from the most disadvantaged socioeconomic positions, particularly among girls.
	Income	• Murayama et al. [116] found that Japanese schoolchildren aged 15–18 y, dietary quality scores were lower for the lowest income level compared with the highest income level.	• Adolescents in poverty tend to consume more unhealthy foods such as sweetened drinks and fast food, as observed by Noonan [115]. • Zagorsky and Smith [118] found that fast-food consumption increased with income from the lowest to middle quintiles, though overall differences were modest, with the wealthiest individuals consuming ∼1 less fast-food meal per week compared with those in the lowest quintile.	• Kurotani et al. [117] found that lower-income individuals aged 15–18 y consumed fewer vegetable dishes, dietary fiber, and potassium compared with their higher-income counterparts. • Murayama et al. [116] found that lower household income was associated with reduced intake of protein-rich foods and green vegetables in Japanese schoolchildren.	—
	Income and education	• Cavaliere et al. [123] showed that both income and education positively impacted adherence to the Mediterranean diet, with education having a more substantial effect. • Manyanga et al. [73] observed that lower socioeconomic status was linked to higher consumption of unhealthy foods among children across multiple countries.	• Miura et al. [119] found that education level, rather than household income, significantly influenced takeaway food consumption patterns in Australia, with those with no post-school qualifications showing a higher prevalence for consuming overall takeaway food >4 times per month compared with those with a bachelor's degree or higher. • Van Kim [109] demonstrated that lower parental education was associated with reduced fruit and vegetable consumption among participants. • Evensen et al. [122] found that individuals with higher educational levels in adulthood and higher educational intentions in adolescence consumed sugar-sweetened beverages less frequently.	• Aggarwal et al. [120] revealed that higher income and education were correlated with lower energy density and higher nutrient adequacy among United States adults, and that the pattern was moderated by education level.	—
	Education and occupation		• Martínez-Martínez et al. [124] found that children's fish intake was significantly associated with their mother's occupation, particularly for lean and fatty fish, whereas the father's occupation and parental education level had no significant impact, leading to recommendations for targeted interventions to address gender disparities in dietary habits.	—	—
	Income, occupation and education	• Patel et al. [126] observed that DASH score improved over time, with the widest socioeconomic differences emerging for consumption of fruit, vegetables, whole grains, nuts, seeds, and legumes.	• Méjean et al. [125] found significant differences in animal foods intake based on education levels, with lower-educated individuals demonstrating higher consumption of red meat, processed meat, and poultry.	—	—
Social capital		No studies explored the role of social capital on dietary inequalities.			
Multiple		• Parker et al. [106] found that high-income females had higher diet quality scores on the AHEI-P compared with middle- and low-income participants, with Non-Hispanic Black females scoring lower than other racial groups.	• Morgan et al. [129] reported that adolescents from lower socioeconomic groups had higher consumption of sugar-sweetened beverages and energy drinks, though there was a 40% reduction in daily SSB consumption from 2000 to 2017. • Zarnowiecki et al. [128] found that income moderated the relationship between boys' fruit and vegetable intake, attitudes, and healthy behaviors, whereas for girls, self-efficacy in healthy behaviors was influenced by occupation rather than income.	• Moore et al. [127] found that high-income participants had significantly greater vitamin D intake compared with medium-income participants, whereas no significant difference was observed for low-income participants, and income disparities persisted among racial groups, particularly for Non-Hispanic Black children in the high-income category. • Kuczmarski [132] observed race-specific differences in flavonoid intake, but did not find significant differences in total flavonoid intake by income.	—

NOTE: Food pattern refers to the overall combination of foods typically consumed, such as the Mediterranean or Western diet. It also includes diet quality indexes such as the Healthy Eating Index and the Dietary Guidelines Adaptation. Food consumption focuses on the specific intake of food groups, like fruits or vegetables. Nutrient intake refers to the amounts of individual nutrients consumed, such as vitamins or minerals [131].

Abbreviations: AHEI-P, Alternative Healthy Eating Index for Pregnancy; DASH, dietary approaches to stop hypertension; PROGRESS, Place of Residence, Race/Ethnicity, Occupation, Gender/Sex, Religion, Education, Socioeconomic Status, and Social Capital; SEP, socioeconomic position; SSB, sugar-sweetened beverages.

**TABLE 2:** Proposed mechanisms for diet-related health inequalities and proposed strategies to mitigate them identified in the sampled studies

Dimension	Mechanisms	Strategies
Place of residence	- Limited access to healthy food options and increased availability of fast food - Poor urban environments - Low perceived control and self-efficacy - Poorer health-related and dietary attitudes - Educational level and family structure - Peer influence and higher income promoting healthier dietary choices - Gender inequalities affecting diet quality scores - Income levels - Employment opportunities - Other regional environmental factors	- Tailor interventions considering individual characteristics and local food environments, especially in vulnerable rural areas. - Improve access to healthy foods for rural residents. - Implement targeted health education campaigns. - Implement government policies to alleviate financial barriers to healthy eating. - Implement targeted intervention programs, such as healthy food vouchers and supplemental food packages. - Improve access to education.
Race/ethnicity	- Acculturation status and cultural factors - Socioeconomic factors like education level, poverty, and household structure - Access to healthy foods, cost of diet, physical access, and marketing strategies - Cultural beliefs, perceptions of health risks, and dietary knowledge - Structural/environmental factors and challenges in upward social mobility - Cultural, religious, and racial/ethnic differences in food accessibility, skills, and time for food preparation, and food insecurity - Cultural heritage and family environment influencing dietary practices	- Encourage healthy snacking and supportive home environments for adolescents, incorporating cultural factors. - Provide culturally tailored dietary counseling. - Advocate for affordable physical activity facilities and equitable access to healthy food outlets. - Propose targeted nutritional interventions and education to improve nutrient intake and reduce unhealthy consumption. - Address discrimination and enhance prenatal nutrition for urban ethnic-minority populations.
Occupation		
Gender/sex	- Social perceptions of “masculinity” and “femininity” influencing eating behaviors and dietary patterns - Gender roles impacting food insecurity and dietary choices	- Interventions targeting gender roles and concepts of masculinity and femininity related to food intake and body.
Education	- Low health literacy - Influence of maternal education on children’s fast food intake - Intergenerational transmission of dietary patterns	- Interventions targeting parental nutritional knowledge and health literacy among adults. - Policy measures aimed at improving educational attainment, particularly among parents.
Religion	- Social norms and religious percepts - Social integration	- Target social marketing strategies such as specialized facilities, worksite programs, printed information.
Social capital		
SES	- Unequal access to resources and cost constraints - Variations in nutritional knowledge - Interaction between nutritional knowledge, socioeconomic factors, and dietary behaviors - Educational attainment influencing occupation and income - Cultural norms shaping dietary decisions - Disparities in nutritional knowledge and economic constraints - Interplay between household nutritional knowledge and genetic factors	- Promote equitable access to resources through national and international policies, considering socioeconomic and gender factors. - Create healthier school environments and provide subsidized school meals for low-income children. - Prioritize sustainable healthier eating habits in policies, addressing socioeconomic disparities with a focus on food affordability and nutrition education. - Use targeted interventions to address dietary disparities and promote healthier eating in urban areas through collaborative public health messaging. - Enhance nutritional knowledge and access to healthy foods among disadvantaged groups through education and affordability initiatives.

Abbreviation: SES, socioeconomic status.

## Discussion

This systematic review provides state-of-the-art evidence on diet-related health inequalities in high-income countries in various age groups. Based on the PROGRESS framework, we offer a comprehensive understanding of how different SDoH modify opportunities and resources toward a healthy dietary intake, identifying possible underlying mechanisms and potential strategies to address them.

The results of this systematic review underscore the existence and reach of diet-related inequalities in high-income countries. Across the various PROGRESS dimensions, individuals in the more disadvantaged groups generally exhibit poorer dietary habits, characterized by lower intake of fruits, vegetables, whole grains, and protein-rich foods, and higher consumption of SSB, fried foods, fast food, and processed meat. When compared with previous reviews on the topic, these findings highlight the persistence of diet-related health inequalities over time [6,60,61]. Social exposures show a significant small to moderate effect size [133], although drawing robust numerical conclusions is not possible due to the heterogeneity of the included studies. The diverse measures, operationalizations, and adjustments of both exposures and outcomes across studies impede direct comparisons and synthesis. This variability highlights the imperative for standardized methodologies both for dietary and sociodemographic and economic variables in future research to enhance the understanding and address the complexities of dietary inequalities.

The mechanisms delineating dietary inequalities within our sampled studies reflect the evidence described in frameworks to conceptualize food security and diet and/or health inequalities [134]. Across various dimensions of the PROGRESS framework, our research findings yield several key conclusions, illustrating both shared patterns and unique factors contributing to disparities. Across almost all dimensions, socioeconomic factors emerge as significant determinants of dietary habits. These findings parallel previous studies [60,61] underscoring the prominent role of SES in shaping dietary habits, not only through a direct effect but also moderating, mediating, and modeling the influence of other dimensions. These findings also align with the evidence on food insecurity, as an epitome of diet-related inequalities, which in high-income countries is primarily driven by an impaired economic access to adequate food and nutrition [135]. The prominent role of SES likely motivates the extensive reviews that focus solely on this axis of inequality [48,51,55]. However, our review highlights that other factors, such as the local food environment, social integration, social norms, and cultural influences, also play a crucial role in shaping diet-related health inequalities, broadening the scope beyond just socioeconomic determinants.

For instance, disadvantaged populations are more likely to be exposed to food deserts and food swamps and when healthy foods are available, they may be out of reach due to financial constraints and transportation barriers [136]. These challenges seem to be exacerbated in rural/remote locations (R) [137]. The influence of occupation on diet is mediated by job-related factors such as work schedules, job security, and stress levels, which can disrupt regular eating patterns and hinder food planning and management [138]. This issue is further compounded by inadequate housing conditions that impair proper food storage and preparation and hazardous living conditions may also complicate family meals and incentivize unhealthy home food environments. Additionally, targeted marketing strategies often promote unhealthy foods, particularly to vulnerable populations [139].

Occupation (not social class/position based on it) and social capital were not investigated in any of the studies in our sample. However, the absence of studies focusing on their influence through the lens of inequalities does not imply a generalized lack of research on these topics. Regarding occupation, although research is limited, some studies have explored how it affects dietary outcomes beyond the social class perspective. For instance, in Japan, Tanaka et al. [140] found significant dietary differences among 38,721 employed fathers-to-be, with security workers consuming more dairy and calcium, and agricultural workers more pickles and salt. Zaganjor et al. [141] found no strong association between occupation and diet quality among pregnant American females, though certain occupations like arts and management were linked to lower diet quality. Both studies suggested that occupation influences dietary habits through various mechanisms beyond SES, including work schedules and job-related stress. Several studies have investigated the association of social capital or social support on dietary habits, finding that it can exert a positive effect on dietary patterns, particularly Mediterranean diet [[142], [143], [144]], but may also have recognized negative health effects [145,146].

The role of social norms is apparent in the case of the gender/sex (G) dimension, with articles discussing how eating behaviors and body appearance are thought of in terms of being masculine or feminine. This influence is also manifest in the case of race/ethnicity (R). The relationship between ethnicity and diet aligns with broader evidence on health inequalities, driven mainly by socioeconomic differences and discrimination processes [147] but with also recognized cultural component, as shown by the significant differences within ethnic groups. Studies on different generations of migrants [97] reinforce this notion, and highlight the importance of cultural heritage and the detrimental effect of acculturation [112]. Although not explicitly addressed in our sample, the adoption of social norms by SES and ethnic groups can be likened to the theory of habitus [148]. For instance, Sato et al. [43] illustrated how food preferences, symbolizing both luxury and necessity, differ across social classes in various countries. Additionally, varying social exposures, such as work characteristics, may further contribute to these differences [149].

Alongside these factors, the studies underscore the influence of psychosocial determinants such as diminished perceived control and self-efficacy, as a consequence of an internalized sense of disadvantage and powerlessness in effecting positive dietary modifications.

Proposed strategies to address dietary inequalities also combine generic and tailored approaches. For place of residence disparities, targeted interventions include community-based initiatives like farmer's markets or mobile food vendors in underserved areas, educational programs to enhance health literacy, and regulatory policies to reduce fast-food establishments and promote fresh produce availability. Social policy and labor market regulations, including housing costs are necessary to address socioeconomic inequalities, with subsidized school meals and other strategies being needed to support the dietary intake of lower-income children and adolescents. Addressing racial/ethnic dietary disparities requires, beyond tackling socioeconomic and discriminatory causes, culturally nuanced interventions, such as tailored nutrition education programs and community-engaged approaches involving local stakeholders. Other strategies proposed in the literature to promote equity in healthy eating include fiscal measures (taxes on unhealthy foods, subsidies for healthy foods, and regulating food advertising), supportive employment policies (promoting healthy eating, flexible work hours), and school-based initiatives (nutrition education, policy changes like removing unhealthy foods) [60,135].

The systematic review is not exempt from limitations. Firstly, the inclusion of observational studies introduces the potential for biases inherent in such study designs, which prevent from establishing causality. However, our choice was motivated toward width of evidence, considering that experimental designs investigating the effect on health of social conditions are scarce and ethically complex [150]. Second, although we provide a systematic examination of the influence of the SDoH on diet inequalities underpinned by the PROGRESS Framework, our review does not include other well-known influences on food intake and potential generators of inequalities such as the food and healthcare systems. Research on these topics is growing [64,151] and a comprehensive analysis is warranted. It is also to note that most of the studies in our sample report data from the United States, potentially limiting the direct extrapolation of conclusions due to political and societal differences. Moreover, we restricted our analysis to the general population, excluding specific groups such as individuals with disabilities and mental health conditions. We also have not explored how diet inequalities may vary or widen among ill individuals. We adopted this strategy to narrow the scope of the review, aiming to facilitate the identification of general trends and interpretations. As suggested by others [47], position paper Dietitians United States and Canada future research should prioritize exploring these interconnected areas using standardized methodologies, as well as assessing the effect of interventions to reduce inequalities across the PROGRESS dimensions, such as family assistance programs, fiscal policies or other political devices.

In conclusion, this systematic review sheds light on the persistent diet-related health inequalities in high-income countries, through the lenses of the PROGRESS framework. Our findings reveal that socially disadvantaged groups face significant barriers to achieving a healthy dietary intake, contributing to widening disparities in diet quality. Individuals from lower socioeconomic backgrounds, as well as those exposed to food deserts, financial constraints, and adverse living and working conditions, consistently exhibit poorer dietary habits. The SDoH, such as SES, occupation, and place of residence, are key factors influencing these dietary patterns, with far-reaching implications for public health.

Addressing these disparities requires comprehensive and targeted interventions that go beyond individual behavior changes and consider broader structural and systemic factors. Policymakers should prioritize the development of strategies such as fiscal measures, community-based initiatives, and regulatory policies that promote equitable access to nutritious foods. Additionally, culturally tailored interventions that address racial, ethnic, and socioeconomic disparities are essential to ensure that all population groups have the opportunity to improve their dietary quality. Improving dietary quality and health outcomes for all population segments requires concerted efforts across multiple fronts, involving policymakers, researchers, and public health practitioners. Urgent action is needed to mitigate diet-related health inequalities.

## Authors contributions

The authors’ responsibilities were as follows – EC-A: conceptualized the study and contributed to data curation and formal analysis; EC-A, MR-M, BS-R, ML: responsible for writing the original draft; and all authors: participated in data curation and formal analysis, contributed to the review and editing of the manuscript, read and approved the final manuscript.

## Data availability

All data generated or analyzed during this study are included in this published article and its supplementary information files.

## Declaration of generative AI and AI-assisted technologies in the writing process

During the preparation of this work, the author(s) used ChatGPT to improve English language writing. After using this tool/service, the author(s) reviewed and edited the content as needed and take(s) full responsibility for the content of the publication.

## Funding

The authors reported no funding received for this study.

## Conflict of interest

The authors report no conflicts of interest.
