# Supplementary material for: Diet-Related Health Inequalities in High-Income Countries: A Scoping Review of Observational Studies
Source: Adv Nutr. 2025 May 5;16(6):100439. doi: 10.1016/j.advnut.2025.100439 (PMC12149430; doi:10.1016/j.advnut.2025.100439)
Supplement: multimedia component 1 [file mmc1.docx]

**Diet-related health inequalities in high-income countries: a scoping review of observational studies**

**Elena Carrillo-Alvarez et al**.

**Supplementary file 1. Search strategy for Pubmed**

("Health Status Disparities"[MeSH Terms]) AND ("Residence Characteristics" [MeSH Terms] OR "Poverty areas" [MeSH Terms] OR "Rural population" [MeSH Terms] OR "Rural health" [MeSH Terms] OR "Urban population" [MeSH Terms] OR "Urban health" [MeSH Terms] OR "Suburban population" [MeSH Terms] OR "Suburban health" [MeSH Terms] OR "Continental Population Groups" [MeSH Terms] OR "Ethnic groups" [MeSH Terms] OR "Culture" [MeSH Terms] OR "Language" [MeSH Terms] OR "Transients and Migrants" [MeSH Terms] OR "Emigrants and Immigrants" [MeSH Terms] OR "Minority groups" [MeSH Terms] OR "Minority health" [MeSH Terms] OR "Occupations" [MeSH Terms] OR "Employment" [MeSH Terms] OR "Unemployment" [MeSH Terms] OR "Gender Identity" [MeSH Terms] OR "Sex" [MeSH Terms] OR "Religion" [MeSH Terms] OR "Education" [MeSH Terms] OR "Educational Status" [MeSH Terms] OR "Social Class" [MeSH Terms] OR "Socioeconomic Factors" [MeSH Terms] OR "Health Status Disparities" [MeSH Terms] OR "Poverty" [MeSH Terms] OR "Income" [MeSH Terms] OR "Remuneration" [MeSH Terms] OR "Salaries and Fringe Benefits" [MeSH Terms] OR "Social mobility" [MeSH Terms] OR "Hierarchy, Social" [Mesh] OR "Social determinants of health" [MeSH Terms] OR "Psychosocial deprivation" [MeSH Terms] OR "Social stigma" [MeSH Terms] OR "Marital status" [MeSH Terms] OR "Social capital" [MeSH Terms] OR "Social conditions" [MeSH Terms] OR "Social environment" [MeSH Terms] OR "Community Networks" [MeSH Terms] OR "Social support" [MeSH Terms] OR "Social isolation" [MeSH Terms] OR "Loneliness" [MeSH Terms] OR "Social alienation" [MeSH Terms] OR "Social marginalization" [MeSH Terms]) AND ("Diet"[MeSH Terms] OR "diet, food, and nutrition"[MeSH Terms] OR "Eating"[MeSH Terms] OR "Feeding Behavior"[MeSH Terms])

| **Supplementary table 1. Simplified extraction table** | | | | | | | | | | | | |
| --- | --- | --- | --- | --- | --- | --- | --- | --- | --- | --- | --- | --- |
|  | **Author, year** | **Country** | **Study design** | **Sample characteristics** | **Age group** | **Objective** | **Exposure** | **Covariates** | **Outcome** | **Results** | **Mechanisms that explain the disparities** | **Possible solutions that are discussed in the paper** |
|  | **Aggarval 2011** | United States | Cross-sectional | 1266 responders (804 women and 462 men). | Adults | To test the hypothesis that diet cost mediates the relation between socioeconomic position and diet quality | SES: Education, income | Age, gender, race/ethnicity, household size, total energy intake | P: Diet quality (mean adequacy ratio, energy density) | Higher income and education were each associated with lower energy density and higher MAR scores; Income–diet cost–diet quality pathway was found to be moderated by education level. | Income directly affects the affordability of foods, influencing diet quality.  Income-diet cost has a stronger impact on diet quality among individuals with lower education compared to those with higher education.  Education not only affects affordability but also reflects an individual's ability to use dietary knowledge to achieve better quality diets within budget constraints. | Strategies to improve diet quality among lower socioeconomic strata may need to take food prices and diet cost along with nutrition education into account. |
|  | **Assari 2020** | United States | Cohort study | 1926 families who were either Black (n = 1429) or White (n = 497). | Households | To test the effect of maternal education on the frequency of eating breakfast, and to compare the effect of maternal educational attainment on the frequency of eating breakfast between Black and White youth. | Ethnicity and education | Youth gender, maternal age at childbirth, family marital status, and family income at baseline were the study covariates | O: Frequency of having breakfast on a weekday | Maternal educational attainment at birth was positively associated with youth frequency of eating breakfast among Whites, not Blacks. We also found a significant interaction between maternal  educational attainment at birth and race, suggesting that the association between maternal education and youth frequency of eating breakfast at age 15 was weaker for Black than White families | Structural/environmental factors, social discrimination, and challenges in upward social mobility. Notably, highly educated Black individuals face stressors and health risks despite their educational attainment. | The proposed solutions involve targeted policies that go beyond improving resource access, emphasizing the need to address Minority/Marginalized Group Discrimination and systemic disadvantages. The study suggests that without tackling discrimination solely enhancing socioeconomic resources may not sufficiently eliminate health disparities |
|  | **Bayram 2018** | Israel | Cross-sectional | 1682 adults | Adults | The objective of this study is to identify factors associated with three health behaviors: physical activity (PA), fruit and vegetable consumption, and smoking taking into account social inequality and religion and ethnicity. | Ethnicity and religion | Age, ethnicity, migrant status, employement status, education and income, health awareness | FC: F/V | Health behaviors are determined by individual characteristics, such as age, ethnicity/religion, economic and cultural resources, and social structure. | Culture/ethinicty, affordability, and gender. For many Jews, acculturation may improve the quality of their diet; however, assimilation of the Arab population to more westernized dietary habits may decrease the quality | Customized information and computer-based information programs; however, the unique characteristics of Ultra-Orthodox Jews, who avoid using computers or watching television, should be considered. Also, small promotional budgets and lack of sustained funding for social marketing from the health sector or the fruit and vegetable industry remain as a barrier to produce long-term changes in intake |
|  | **Bekelman, 2020** | United States | Cross-sectional | 3636 Mexican American and non-white hispanic adolescents | Adolescents | This study compared diet quality and snack intakes among non-white hispanic and Mexican American adolescents from different acculturation groups. | Ethnicity | Child sex Ratio of family income to poverty (PIR) | P: HEI FC: snack intake | Snack portion sizes and the contribution of snacks to total daily energy intake were substantial and were greater among NHW compared to Mexican American adolescents from most acculturation groups. | The association between acculturation and diet likely results from changes in culture and environmental conditions that accompany integration into a high-income, industrialized country | Minimizing the effects of acculturation on adolescent nutritional health should include support for healthier snacking habits. Specifically, increasing the proportional contribution of fruits, vegetables, and protein-rich foods to snacking occasions while decreasing the proportional contribution of energy-dense snacks and sweets and beverages may lead to improvements in overall diet quality and reductions in obesity risk. |
|  | **Bekelman 2021** | United States | Cross-sectional | 482 children aged 5 | Children | To assess adherence to the 2015-2020 Dietary Guidelines for Americans and identify sociodemographic predictors of adherence among children. | Multiple | Sex, race/ethnicity, maternal education, maternal employment, maternal subjective social status and household income respectively for each exposure | P: HEI | In the full sample, 29% of children were non-adherent to PA and dietary guidelines, 6% adhered to the dietary guidelines only. Girls had 41% lower odds of adhering to the physical activity guidelines than boys, after adjustment for race/ethnicity, household income and maternal education level, perceived social status and employment status. | NA | NA |
|  | **Evensen 2018** | Norway | Cohort study | 437 Participants, 55% female- Data from the first survey in 2001 (mean age 11.8) and follow-up surveys in 2005 (mean age 15.5) and 2016 (mean age 26.5) | Youth (11.8-26.5) | To analyze; (i) the development in frequency of consumption of sugar-sweetened beverages (SSB) and artificially sweetened beverages (ASB) from childhood to adulthood; (ii) socioeconomic inequalities in the consumption of SSB and ASB using different indicators of socioeconomic status (SES); | SES: Education, income | School as random effect, and time, gender, grade level and the different indicators of SES as fixed effects | FC: SSB, ASB | Participants with a higher educational level in adulthood and higher educational intentions in adolescence had a significantly lower frequency of consumption of SSB at all time points (2001, 2005 and 2016). No significant widening (or narrowing) of inequalities were observed from childhood to adulthood. | Diet cost, nutritional knowledge, educational prospects, home environment, effect of public policy. | Structural measures (increased tax on non-alcoholic beverages, reduced food marketing to children, school meal recommendations) |
|  | **Brunst 2013** | United States | Cross-sectional | 274 pregnant women | Pregnant women | To assess sociodemographic correlates of micronutrient intakes from food and dietary supplements in an urban, ethnically diverse sample of pregnant women in the United States. | Ethnicity, education, income | Age, BMI, smoked during pregnancy | NC: vitamin A, C and E, magnesium, selinium, zinc, vitamin B12 and B6, folate, riboflavin, iron, choline, vitamin D, calcium, phosphor, potassium, sodium, thiamin, niacin and vitamin K | Factors associated with multiple antioxidant inadequacies included being Hispanic or African American, lower education and self-reported economic related food insecurity. Hispanics had a higher prevalence of multiple methyl-nutrient inadequacies compared with African Americans; both had suboptimal betaine intakes and higher odds for vitamin B6 and Fe inadequacies compared with Caucasians. Nearly all women (98 %) reported Na intakes above the tolerable upper limit; excessive intakes of Mg (35 %), folate (37 %) and niacin (38 %) were also observed. | NA | Prenatal nutritional interventions may be enhanced in urban ethnic-minority populations if tailored to account for the specific sociodemographic determinants of prenatal dietary patterns. |
|  | **Carroll-Scott 2013** | United States | Cross-sectional | 1048 fifth and sixth grade students | Children | Examine associations between built, socioeconomic, and social characteristics of a child’s residential environment on body mass index (BMI), diet, and physical activity. | Neighborhood environment characteristics | Socioeconomic variables (Black, Latino, female, school free/reduced lunch eligibility), neighborhood socioeconomic variables (percent Black, percent Latino, concentrated affluence, concentrated disadvantage), and school clustering | FC: Healthy foods (F/V wholegrains, nuts, beans) Unhealthy foods (fast food, salt, fat, SSB) | Distinct domains of neighborhood environment characteristics were independently related to children’s health behaviors. Findings link healthy behaviors with built, social, and socioeconomic environment assets (access to parks, social ties, affluence), and unhealthy behaviors with built environment inhibitors (access to fast food outlets), suggesting neighborhood environments are an important level at which to intervene to prevent childhood obesity and its adverse consequences. | Neighborhood environments: easy access to stores and  restaurants selling unhealthy items, normative culture that promotes healthy lifestyles | To effectively address the current obesity pandemic, our findings reinforce the need for sustainable, locally meaningful programs and policies that go beyond individual-level behavior change approaches to provide safer, healthier neighborhood environments for children to thrive and grow into healthy adults. |
|  | **Cavaliere 2019** | Italy | Cross-sectional | 36,032 individuals (adults older than 18 years old). | Adults | Investigate the role of the main socioeconomic and demographic factors in affecting the consumption frequency of specific food categories, and analyze whether socioeconomic status (SES) is related to overall adherence to Mediterranean Diet (MD) of the Italian population. | Multiple: occupation, sex, educational status, income, marital status, age and household size | Education, income, age, gender, household size and marital status | P: Mediterranean diet (ad hoc index) | - More affluent people consume more fish, fruit and vegie, wine and beer, and less meat and eggs, dairy products, cereals and starchy vegetables as well as legumes. - Younger people consume meat, fish, snacks, soft drinks, alcoholic beverages and water more than older segments of the population. - Less educated and lower income people show less adherence to Mediterranean Diet. | Nutritional knowledge, nutritional awareness and dietary costs. | Policy interventions that facilitate availability and affordability of healthy food items |
|  | **Chzhen 2018** | 34 countries | Cohort study | 700 000 15- year-old adolescents in 34 countries | Adolescents | Investigated the role of socio-economic factors as potential determinants of bottom-end health behaviours pertaining to physical activity and diet. | SES: Socioeconomic status (FAS scale) | Age, gender | P: Healthy eating (F/V) & unhealthy eating pattern (sweets, SSB) | Adolescents from less affluent families were much more likely to report being in the bottom-end of the distribution of these health indicators. Large, persistent and widespread socio-economic gradients existed for physical activity and healthy eating, while the findings were mixed for unhealthy eating. Such socio-economic inequalities were largely stable or widened for physical activity and healthy eating, while inequalities in unhealthy eating narrowed. | Nutritional knowledge, income, taste of luxury | Public health interventions focused on physical activity  and diet, including social policies that specifically target these and  other aspects of health in our most disadvantaged children, as priorities, nationally and internationally. |
|  | **Esquius 2021** | Spain | Cross-sectional | 7319 adolescents | Adolescents | To explore the relationships between breakfast and sociodemographic characteristics, health-related behaviors, and school performance of 7319 adolescents. | SES: Socioeconomic position (subjective) | Health and social variables (on a diet, BMI, pha, emotional state, general health status, academic performance, municipality size, migratory status) | O: Breakfast consumption | The prevalence of skipping breakfast every day was 19.4% in girls and 13.7% in boys and was related to students’ SEP. The risk of skipping breakfast was 30% higher in girls from the most disadvantaged SEP, in comparison to those in the most advanced SEP | Different parental expectations, the availability of educational resources at home, or the influence of the socioeconomic position of peers | Future public policies should be adapted considering a SEP and gender perspective to avoid increasing nutritional and health inequalities. |
|  | **Friis 2016** | Denmark | Cross-sectional | 29,473 participants 25 years or older | Adults | Investigate whether health literacy mediates the association between educational attainment and health behavior (smoking, physical inactivity, poor diet) and obesity. | SES: Education | Age, gender, ethnic background, and marital status | P: Dietary Quality Score | The study showed that health literacy in general and the ability to understand health information in particular mediated the relationship between educational attainment and health behavior, especially in relation to being physically inactive (accounting for 20% of the variance), having a poor diet (accounting for 13% of the variance), and being obese (accounting for 16% of the variance). | Social norms, workplace environments, knowledge, stressors/ resources, and work status/income | Interventions aimed at improving health behavior and health status have the potential to become more targeted and effective when informed by robust data on the health literacy of the target populations. |
|  | **Guerrero 2015** | United States | Cross-sectional | 15,902 children aged 2 to 11 years, female 49.2% | Children | To examine the racial and ethnic differences in reported dietary practices among the largest minority groups of California children. | Ethnicity | Income, education, Age, sex, BMI, single-parent household status, household size, caregiver age, caregiver sex, survey year | FC: F/V 100% juice SSB Sweets Fast food | In multivariate regressions, substantial differences in fruit juice, fruit, vegetable, sugar-sweetened beverages, sweets, and fast-food consumption were found among the major racial and ethnic groups of children. Asians regardless of interview language were more likely than whites to have low vegetable intake consumption and low fruit consumption. Latinos regardless of interview language were also more likely than whites to have high fruit juice. Latinos were less likely than whites to consume sweets. | Nutritional knowledge, acculturation, difficulty finding traditional hat are native to the country of origin, and cultural and native patterns where low fruit consumption may be typical. | Increased fruit and vegetable consumption appears to be associated with parent education but not income.  Anticipatory guidance and dietary counseling might benefit from tailoring to specific ethnic groups to potentially address disparities in overweight and obesity. |
|  | **Haughton 2016** | United States | Cross-sectional | Hispanic 608, non-Hispanic black 609, Asian 253, and non-Hispanic white 484 6-19 y.o | Children Adolescents | To evaluate racial/ethnic disparities among children and adolescents in meeting the 4 daily 5-2-1-0 nutrition and activity targets in a nationally representative sample. | Ethnicity | Child BMI percentile and sociodemographic characteristics (age, sex, parental maritual status, household income-to-poverty ratio, parental education) | FC: F/V SSB | None of the adolescents and <1% of children met all 4 of the 5-2-1-0 targets, and 19% and 33%, of children and adolescents, respectively, met zero targets. No racial/ethnic differences in meeting zero targets were observed among children. Racial/ethnic differences in meeting individual targets were observed among children and adolescents. | Racial/ethnic disparities  Childhood adverse events (eg, abuse, maltreatment), which have been shown to be associated with increased risk of overweight and obesity and disordered eating behaviors later on in life. | Renewed and expanded policies targeting obesity, such as regulations and taxes on sugar-sweetened beverages, may be needed to change social norms and the obesogenic environment. Programs supporting youth in meeting dietary and physical activity guidelines, especially by limiting sugar-sweetened beverages and increasing fruit and vegetable consumption, are essential to address and prevent obesity and related behaviors at the population level. |
|  | **Hauschildt 2019** | United States | Cohort study | 3,617 adults ages 25 and up | Adults | Health behaviors are seen as one possible pathway linking race to health outcomes. Social integration has also been consistently linked to important health outcomes but has not been examined as a mechanism accounting for racial differences in health behaviors among older U.S. adults. | Ethnicity | Model 3 is an ordered logistic (linear probability for drinking) model including race, gender, age, SES, health status, and discrimination; Model 4 is Model 3 + all social integration measures (employment status, marital status, and church attendance) | FC: F/V, fried foods, olive oil, whole grain breads and cereals, and red meat | We find differences by race and social integration measures in dietary behaviors and alcohol use. Net of socioeconomic status, health status, and reported discrimination, variation in social integration helps to account for racial differences in some health behaviors | Social Isolation (versus informal integration, church attendance, being married).  Groupal environment to share cultural practices that are linked to positive dietary habits. | NA |
|  | **Highland 2016** | United States | Cross-sectional | 203 latina and nonlatina mothers with children under the age of 18 | Children | To identify ethnic differences in parental health beliefs and their relation to children’s health behaviors (SSB, fried foods). | Ethnicity | Age, birth country | FC: SSB, fried foods | Children of Latina mothers consumed significantly more soda and fried foods and exercised less than children of non-Latina mothers. Latina mothers were significantly more likely to perceive barriers to healthy eating and significantly less likely to perceive benefits to healthy eating and physical activity than non-Latina mothers. Ethnicity mediated the relationship between maternal views of health benefits and soda consumption. | Parental unawareness of the risks of soda consumption.  Influence of parental beliefs on children's soda intake.  Lack of cultural significance of soda.  Mediation of healthy eating beliefs in the relationship between ethnicity and soda consumption. | Policies to address the lack of safe green spaces and access to healthy foods in primarily low-income, Latino neighborhoods. Actions targeting maternal believes. |
|  | **Kurotani 2021** | Japan | Cross-sectional | 866 household members (435 men and 431 women) // aged 6–18 years old //the 2014 National Health and Nutrition Survey, Japan | Children Adolescents | To examine the association between diet quality and household income level in school children in Japan according to age subgroups | SES: Income | Age, sex, residential block, population size of residential area, household size, and current smoker in family members | P: Dietary Quality Score | In this cross-sectional study in Japan, among those who aged 15–18 years old, individuals with the lowest household income level had a lower quality diet compared to individuals with the highest household income level, whereas, those who aged 6–14 years old (who mostly received school lunches) had no significant association between diet quality and household income level. | Income-cost of diet. | To reduce diet disparities by socioeconomic status among high school students, further studies are needed to develop interventions providing school lunches or affordable and nutritious food at a low price in school canteens.  Food and nutrition education programs. |
|  | **Larson 2015** | United States | Cross-sectional | 2374 adolescents (53.8% girls) who completed the EAT 2010 survey | Adolescents | To examine ethnic/racial differences in the home/family environments of adolescents and associations with dietary intake and weight status | Ethnicity | Gender and age, and parental education | P: Dietary Intake | Parental encouragement for healthy eating was associated with lower intake of sugar-sweetened beverages only among youth representing the White, African American, Asian, and mixed/other ethnic/racial groups and was unrelated to intake among East African, Hispanic, and Native American youth | Characteristics of a supportive home/family environment were linked to indicators of better dietary intake in adolescents despite variation in the relevance of specific characteristics across outcomes | Recommendations for providing a home environment that promotes healthy eating and reduces risk for obesity// health programs and services for adolescents should encourage diverse parents to follow existing recommendations |
|  | **Lee 2019** | United States | Cross-sectional | Data from the National Longitudinal Survey of Youth 1979 Children and Young Adult// 3,115 non-Hispanic White males, 1,617 African American males, and 1,144 Hispanic males | Adults | To examine the impact of educational attainment on health behaviors across young adult men, and differences in the association across race/ethnicity | Ethnicity and education | Age (ranging from 18 to 35), family size, married | FC: F/V, fast food | Individuals who received higher levels of education were more likely to show health behaviors in both food intake and preventive health care visits. Furthermore, interaction effects of race/ethnicity were found: African Americans moderated the relationship between educational attainment and food intake (fast food and fruit intake). | Higher education provides more exposure to health information, leading to informed dietary choices and overall lifestyle. It also promotes positive perceptions and attitudes towards health.  Education increases the likelihood of employment in jobs with better conditions, allowing focus on personal health.  Educated individuals often live in areas with better access to fresh produce and fewer fast-food outlets.  Moreover, there may be influences by other SES aspects, like income, influencing the relation through diet cost.  Racial/ethnic disparities affect the relationship between education and diet; for example, African American men face economic and environmental challenges despite higher education. | Education, especially for African American, should provide content improve health behaviors and to lessen racial/ethnic disparities in health behaviors |
|  | **Lee 2020** | United States | Cohort study | Young women includes 2,993 non-Hispanic White, 1,574 African American, and 1,071 Hispanic/Latina women | Young adults | To understand the intergenerational relationship between mother and daughter and the effect of mothers’ education on their young adult children’s fast food consumption. | SES: Education | Age, marital status, and income, daughteers' education | FC: Fast food | mothers’ educational attainment significantly influences their  young adult daughters’ fast food intake, in that young women whose mothers received higher education were less likely to eat fast food. In addition, moderating effects of race/ethnicity were found. Being African American or Hispanic/Latina moderated the relationship between mothers’ educational attainment and their young adult daughters’ fast food intake. | Educated mothers have more knowledge about healthy food and influence their children. Higher education often leads to better-paying jobs, enabling families to afford healthier food options instead of cheaper fast food. African American and Hispanic/Latina women face job discrimination and lower wages even with higher education, leading to economic hardships that force reliance on cheaper fast food. These racial/ethnic groups are more likely to live in areas with many fast food restaurants, increasing fast food consumption despite higher maternal education. | Providing more opportunities for mothers to increase their educational attainment should be considered |
|  | **Liu 2021** | United States | Cohort study | 20 905 children 5 to 19 years of age// 39 757 adults 20 years or older// National Health and Nutrition Examination Survey cycles (2003-2018) -NHANES// (5-11, 12-19, 20-49, and ≥50 years) | All ages | To examine current levels and historical trends in total and subtypes of junk food consumption, focusing on foods (rather than ssbs) and their food sources overall and in population subgroups among nationally representative samples of US children and adults | Ethnicity, education, income | Age, marital status, birth country | P: HEI | Diet quality of foods consumed from grocery stores increased modestly in children (53.2% to 45.1% with poor diet quality) and adults (40.1% to 32.9% with poor diet quality)// disparities in diet quality trends were seen by sex, race/ethnicity, educational level, and household income for food consumed from grocery stores. | Economic and residential barriers to accessing healthy food choices. | The retail grocery environment remains a top opportunity for improving diet quality, followed by restaurants, schools, and, increasingly, other  settings, such as entertainment venues and food trucks. Results support testing and scaling new approaches to positively guide  consumer choices in retail settings, including online shopping. Worksite nutrition is also an opportunity to be promoted. |
|  | **Livingstone 2017** | Australia | Cross-sectional | Adults (≥19 years; n = 4875) collected during the National Nutrition and Physical Activity Survey (NNPAS) component of the 2011-13 Australian Health Survey. | Adults | To investigate associations between SEP and dietary ntakes and to evaluate how these relationships differ by sex, in a nationally representative sample of Australian adults | SES: SEP (area-level disadvantage, education level, and income) |  | P: Dietary Guidelines Intake NC: Nutrient intakes (total energy (KJ/day), percentage energy from total fat, saturated fat (SFA), mono-unsaturated fat (MUFA), poly-unsaturated fat (PUFA), trans fat, carbohydrates, total sugars and protein, and fibre and sodium density | Lower socioeconomic position (SEP) was associated with lower diet quality and poorer intake of certain foods and nutrients among Australian adults. Specifically, lower SEP was linked to a 2.5–4.5 unit decrease in diet quality index (DGI) scores, depending on the SEP indicator. Greater area-level disadvantage correlated with up to a 3.6 unit decrease in DGI scores, affecting adherence to dietary recommendations for 6 out of 13 components. Individuals with lower education had up to 4.5 units lower DGI scores and poorer adherence in 9 out of 13 components. Lower income was associated with up to 2.5 units lower DGI scores and poorer adherence in 6 out of 13 components.  The article also discusses differences in nutrient intake. | NA | Develop targeted interventions that address dietary behaviors across all socio-economic levels, particularly focusing on lower SEP groups, and to address the underlying social determinants of health inequities for broader improvements in dietary behaviors |
|  | **Luk 2018** | United States | Cross-sectional | 1,926 adolescents who participated in the NEXT Generation Health Study in 2010–2011 | Adolescents | To examine sexual orientation differences in eating behaviors, physical activity, and weight status among adolescents | Gender & Sexual orientation | Age, BMI | FC: F/V, SSB, Snacks, whole grains. | Sexual minority adolescents consumed more fruits and vegetables than heterosexual peers, and extended their findings by demonstrating no sexual orientation differences in snack, sugar-sweetened soda, or whole-grains consumption | Sexual minorities may be more sensitive to societal norms regarding beauty, specifically regarding thinness. | NA |
|  | **Lutfiyya 2012** | United States | Cross-sectional | Behavioral Risk Factor Surveillance Survey (BRFSS); 8,983,840 (rural); 18 to >65; caucasian, african american, hispanic, other | Adults | To examine the prevalence differences between US rural and non-rural adults in consuming at least five daily servings of combined fruits and vegetables | Rural/Urban | Age, marital status,children in Household, BMI, Physical Activity, Have Health Insurance, Timing Of Last Routine Medical Checkup; Deferment Of Medical Care Because Of Cost; Self-Defined Health Status | FC: F/V | Rural adults were less likely to consume five or more daily servings of fruits and vegetables (OR = 1.161, 95% CI 1.160-1.162). Logistic regression  analysis revealed that US rural adults consuming at least five daily servings of fruits and vegetables were more likely to be female, non-Caucasian, married or living with a partner, living in a household without children, living in a  household whose annual income was > $35,000, and getting at least moderate physical activity. | limited access to affordable healthy food due to fewer stores offering diverse options, often resulting in food deserts. Economic factors play a significant role, as rural residents typically have lower incomes, making it harder to afford healthy foods. Additionally, food costs are generally higher in smaller markets compared to larger supermarkets, which are often less accessible. Transportation barriers further complicate access, as residents may struggle to travel longer distances to find better food options. | Strategies aimed at improving access to healthy foods for rural residents. |
|  | **Manyanga 2017** | 12 countries (Australia, Brazil, Canada, China, Colombia, Finland, India, Kenya, Portugal, South Africa, the United Kingdom and the United States of America) | Cross-sectional | International Study of Childhood Obesity, Lifestyle and the Environment (ISCOLE); 8,808; children 9-11; urban/peri-urban sites | Children | To examine relationships among dietary patterns and SES of children from countries spanning a wide range of human development | SES: Education, income (combined) | Age, BMI | P: Dietary Quality Score | At all levels of  country-level human development, lower income or lower levels of parental education were associated with higher consumption of unhealthy foods (higher UDP  scores) and lower consumption of healthy foods (lower  HDP scores). | Unhealthy foods lower cost (higher affordability) when compared to healthy foods. | Implementing community initiatives and support for local markets. Increasing the availability of fresh, healthy food options can help mitigate the reliance on processed foods. Additionally, strengthening nutrition education programs can empower lower-income households, thereby reducing disparities in dietary quality across socio-economic groups. |
|  | **Martin, C.L. 2017** | United States | Cross-sectional | 282; women >18 | Adults | To examine the associations between acculturation (US culture in mainly mexican women) and maternal diet, physical activity, and post-partum weight retention (PPWR). | SES: Education (acculturation) | Months postpartum at study entry, maternal age at study entry, breastfeeding status, education level, parity, and total energy intake, prepregnancy BMI as well as the diet and physical activity variables that were significantly associated with acculturation. | P: HEI  FC: F/V, whole grains, Fast food  NC: Energy intake, Total fat, Total protein, Total carbohydrates, Saturated fat, Percent energy intake from fat, Percent energy intake from protein, Percent energy intake from carbohydrates, | More acculturated women had lower intakes of fruits and vegetables, lower HEI scores, and lower physical activity levels than women who were less acculturated (p < 0.05). We found an association between acculturation and PPWR in that for every 1-unit increase in acculturation score, PPWR increased, on average, by 0.80 kg |  | NA |
|  | **Martin, J.C. 2017** | Australia | Cross-sectional | Healthy Lifestyles (help-her) cluster randomized controlled trials; 543; women; 18-50 | Adults | To compare diet quality in urban and rural women of reproductive age, and secondary analyses of the difference in macronutrient and micronutrient intake in urban and rural women, and the predictors of diet quality. | Rural/Urban | Education, income, working, body mass index, age, marital status and town clustering | P: Dietary Guideline Index (DGI) NC: Energy, Protein, Carbohydrates, Fat, Sat Fat, Mono Fat, Poly Fat, Fibre, Cholesterol, Glycemic Index, Glycemic Load, Calcium, Iron, Folate, Sodium | No significant difference in diet quality between cohorts (urban vs rural); predictors of diet quality and report that higher income levels and working status are associated with better diet quality in all women of reproductive age | Cost and affordability of healthy food, and intrahousehold coping strategies before food insecurity. | Women who are unemployed and on a lower income are an important target group for future dietary interventions aiming to improve diet quality. |
|  | **Martínez-Martínez 2020** | Spain | Cross-sectional | 131 children of both sexes | Children | To assess the effect of the intake of common foods containing high amounts of omega-3 polyunsaturated fatty acids | SES: Education and occupation | Age, BMI | FC: Fish NC: Omega-3 long-chain polyunsaturated fatty acids | There was no significant association between the father’s type of work and the children’s fish intake, except for shellfish intake. However, there were significant associations between the mother’s work and the children’s fish intake. No significant associations were observed between parental level of education and fish intake in children | Social aspects, such as the type of work done by mothers and their educational levels are significant factors affecting children’s/adolescents’ intake of DHA+EPA. | Dietary interventions to increase the consumption of fish and seafood are strongly advised, and health promotion strategies should be aimed at the family level and fight against gender disparities. |
|  | **Maruyama 2017** | Japan | Cross-sectional | 1,447; 10-11; children (plus guardian) | Children | To examine the association between household income and the intake of foods and nutrients by Japanese schoolchildren, and any differences between days with and without school lunch | SES: Income | Physical activity, food allergies/food restrictions, BMI | FC: Rice, noodles, breads, cereals, Potatoes, Sugar, Nuts and seeds, Green vegetables, Other vegetables, Fruit, Mushrooms, Seaweeds, Beans, Fish and shellfish, Meats, poultry, Egg, Milk, dairy products, Fat and oil, Confectionery, Drinks NC: Energy, Protein, Animal protein, Lipids, Saturated fat, Cholesterol, Carbohydrate, Dietary fibre, Energy from protein, Energy from fat, Energy from carbohydrate, Retinol activity equivalents, Vitamin D, α-Tocopherol, Vitamin K, Thiamin, Riboflavin, Niacin equivalents, Vitamin B6, Vitamin B12, Folate, Pantothenic acid, Biotin, Vitamin C, Na, K, Ca, Mg, P, Fe, Zn, Cu, Mn, Iodine | The results indicate that children from low-income households consume lower levels of protein and micronutrients and a higher percentage of energy from carbohydrates due to reduced intake of protein foods and green vegetables, relying more on staple foods. Additionally, the study found no significant disparities in food and nutrient intake between income groups on school lunch days, suggesting that the school lunch program effectively reduces dietary disparities related to household economic status. | The association between household income and dietary intake is influenced by food costs, as nutrient-dense foods are typically more expensive than energy-dense options. Higher food costs correlate with increased intake of healthy foods, while lower costs are associated with higher consumption of fats and sugars. | food costs mediate the relationship between household income and children's dietary intake, suggesting that improving economic factors could reduce dietary disparities among Japanese children. School lunch programs may contribute to reduce food insecurity. |
|  | **McCartney 2013** | Ireland | Cross-sectional | 292; women; 18-35 | Adults | To investigate socio-economic disparities in food and nutrient intakes among young Irish women | Multiple: area level, educational attainment and occupational social class, in addition to material indices of disadvantage including ‘at risk of poverty’ status, relative deprivation and consistent poverty |  | FC: F/V, breakfast cereals, dairy products, meat and meat products, starchy carbohydrates, sweet foods and drinks, potatoes, fish and fish products. NC: Dietary fibre (Southgate), % Food energy from carbohydrate, % Food energy from non-milk extrinsic sugars, % Food energy from fat, % Food energy from saturated fat, Cholesterol, Alcohol (units/week), Vitamin B1, Vitamin B2, Niacin, Vitamin B6, Vitamin B12, Folate, Vitamin C, Vitamin A, Vitamin D, n-3 PUFA, Na, Fe, Ca, Zn, Cu, P, Energy (kcal), Energy (MJ), NSP (Englyst), Carbohydrate, NMES, Total fat, Saturated fat, Monounsaturated fat, Polyunsaturated fat, Protein, Vitamin B5, Carotene, Vitamin E, K, Mg. | The study reveals that young, socially disadvantaged women in Dublin have less favorable dietary habits compared to their more affluent peers, characterized by lower intakes of dietary fiber and essential micronutrients, higher intakes of fats and processed foods, and a diet low in fruits, vegetables, whole grains, and fish. This pattern reflects the prevalence of low-cost diets that contribute to significant nutrient inadequacies within this group. | Putative barriers to healthy diet among low-SES groups include poor nutritional knowledge, inadequate food preparation skills, high cost of healthy food, poor local food environment, low perceived control and self-efficacy and poorer health-related and dietary attitudes | Public health interventions targeting low-income women, with a focus on increasing the intake of healthy foods, with the displacement of less healthy alternatives being a key objective- |
|  | **Méjean 2016** | France | Cross-sectional | Nutrinet-Santé Study; 92,036; men and women; >18 | Adults | The specific role of major socio-economic indicators (education, occupation, income) in influencing consumer choice of animal foods (AF) intake could reveal distinct socio-economic facets, thus enabling elucidation of mechanisms leading to social inequalities in health. We investigated the independent association of each indicator with intake of different AF and their effect modification | SES: Education, occupation, income | Age, total energy intake, BMI, total animal foods intake, occupation, household income and education | FC: Animal food group intakes | t low socio-economic  populations, particularly in terms of education, made  unhealthier AF intake choices than persons in higher categories; these included meat products and high-fat dairy desserts instead of fish and low-fat desserts. In addition,  simultaneous use of three socio-economic indicators and the study of their interactions highlighted distinct facets of SEP that may influence AF intake. Specifically, Education  appears to be the strongest and most robust independent predictor of AF intake | Education significantly influences dietary choices, with higher education levels leading to greater awareness of health risks and reduced meat consumption. Economic factors also play a role, as lower-income individuals often opt for cheaper, less nutritious foods, resulting in lower fish intake. Social norms can reinforce meat consumption among less-educated individuals, while cultural practices ensure consistent cheese intake across socio-economic groups. Additionally, dietary habits formed in childhood often persist into adulthood, impacting long-term nutrition. | Our findings provide information useful for identifying subgroups of the population at high nutritional risk in terms of AF intake. This is a key element when implementing nutritional public health measures targeting disadvantaged groups, particularly in the current context of health inequalities, which remain important; Understanding why persons with less education prefer eating meat is critical, since they are more strongly affected by chronic diseases for which meat intake is a risk factor |
|  | **Mendez 2019** | United States | Cohort study | What We Eat in America, the dietary component of the NHANES surveys; 17,579; children; 2-18 | Children | To estimate usual SSB intake from NHANES surveys from 2003–2004 to 2013–2014 to examine shifts at both the median and 90th percentile among US children, evaluating the extent to which intake disparities in total ssbs and subtypes have persisted | Ethnicity | NHANES cycle year, age group, sex, race/, parental education, and per capita household income as a percentage of the FPL, weekend (including Friday), as well as season of data collection, self-reported intake amount, and recall sequence. | FC: SSB | There were important disparities in SSB intake, with considerably higher usual intakes in several vulnerable groups, including adolescents and children from lower-income households. Interestingly, racial/ethnic disparities in total SSB intake varied by income group. A strong income gradient in SSB usual intake was apparent among NHW children and to a lesser extent among mas, but SSB usual intake among NHB children was similarly high across households of diverse income levels. | Targeted marketing strategies to vulnerable populations, including children. | Targeted public policy. Sstrategies to  reduce heavy consumption of SSBs among children adequately  reach socioeconomically diverse NHB children as well as lowerincome households. SSBs taxes may be effective. |
|  | **Miura 2012** | Australia | Cross-sectional | Men and women; 25-64 | Adults | To examine socio-economic differences in the frequency and types of takeaway foods consumed | SES: Education, income | Age, sex | FC: Takeaway foods | Compared with their more educated counterparts, the least educated were more regular consumers of overall takeaway food and fruit or vegetable juice and less regular consumers of sushi. For the ‘less healthy’ items, the least educated more regularly consumed potato chips, savoury pies, fried chicken and non-diet soft drinks; however, the least educated were less likely to consume curry. Household income was not associated with overall takeaway consumption. The lowest-income group was a more regular consumer of fruit or vegetable juice compared with the highest-income group. Among the ‘less healthy’ items, the lowest-income group was a more regular consumer of fried fish, ice cream and milk shakes, whereas curry was consumed less regularly. | as income influences the affordability of healthier food options, while education impacts nutritional awareness and dietary choices. Cultural norms also shape preferences, such as higher fruit drink consumption among non-Hispanic Black children. Additionally, disparities in access to healthy food environments, food marketing targeting lower-income communities, and varying levels of health literacy further contribute to these differences. Together, these factors create a complex interplay that influences dietary behaviors across socioeconomic and racial/ethnic groups. | NA |
|  | **Moore 2014** | United States | Cross-sectional | NHANES; 6,226; children; 1-18 | Children | To estimate vitamin D intakes of children 1 to 18 years old in the United States by race/ethnicity, sex, age, and family using 24-hour dietary intake recalls and dietary supplement use questionnaires | Multiple: race/ethnicity, sex, age, and family income | Age | NC: Vitamin D | Total, dietary, and supplement intake of vitamin D of all participants were significantly greater in the high-income vs the medium-income group. Although mean total vitamin D intake of the low-income group was less than the high-income category, the difference was not significant. NH whites had a significantly higher total intake of vitamin D than NH blacks and Hispanics; total vitamin D intake of Hispanic children was significantly greater than that of NH black children; When groups were compared by income category across race/ ethnicity, total vitamin D intake of NH whites was greater; than that of NH blacks for the high-income category, but not compared to the medium-income category | Although vitamin D–fortified milk is relatively inexpensive per serving, the cost of plant-based milk (ie, fortified soy milk) and enriched mushrooms containing vitamin D may prevent lower economic groups from choosing these foods | NA |
|  | **Morgan 2021** | United Kingdom | Cohort study | 176,094 children; 11-16 in Health Behaviour in School-aged Children (HBSC) survey and the Welsh School Health Research Network (SHRN); | Children | To date no study has examined time trends in adolescent consumption of sugar-sweetened beverages and energy drinks, or modelled change in inequalities over time. The present study aimed to fill this gap by identifying historical trends among secondary school students in Wales, United Kingdom. | Multiple: sex, SES (FAS) | Age | FC: SSB and Energy Drinks | We found that consumption reports since 1998 indicate a positive shift for daily SSB consumption with approximately 40% fewer adolescents reporting daily consumption in 2017 compared to 2000; We found clear patterning of SSB and ED consumption according to SES, observing higher consumption rates among young people from lower socioeconomic groups | Public policy targeting SSB may be responsible for the decline in SSB consumption, but cost and targeted marketing could be widening SES disparities | Pinpointing the underlying factors which contribute to such socioeconomic differences is key to  ensuring policy interventions facilitate healthy food choices for all population groups |
|  | **Newby 2010** | United States | Cross-sectional | 9559 men | Adults | To examine dietary intakes of men currently enrolled in the Reasons for Geographic and Racial Differences in Stroke (REGARDS) study and determine whether there were racial and regional differences in intakes among black and white men living in the Stroke Belt compared with those living elsewhere in the US. An additional goal was to examine whether race and region were significant predictors of selected nutrient intakes and whether the 2 would interact. | Ethnicity and residence characteristics | Age, total energy, BMI, multivitamin use, income, education, marital status, smoking status, alcohol use, physical activity, television viewing, and diagnoses of disease (hypertension, hypercholesterolemia, and diabetes) | NC: Energy, Carbohydrate, Protein, Total fat: Saturated, Trans, Monounsaturated and Polyunsaturated, Fiber, Alcohol | Compared with white men, black men within each region consumed a higher percentage of energy from carbohydrate and a lower percentage of energy from total fat; intakes of fiber and alcohol were also lower for black men .lack men consumed a lower percentage of energy from trans fat compared with white men in the Stroke Belt but not in the Stroke Buckle White men had a higher median intake of total energy in the Stroke Belt and other regions only ;total energy intake did not differ across regions. Black men consumed lower median intakes of riboflavin, niacin, vitamin B-12, a-tocopherol, vitamin D, calcium, potassium, magnesium, sodium, and iron compared with white men within each region. Black men consumed more vitamin C than white men in the Stroke Belt and Buckle but intakes in the other regions did not differ | Race and region were significant predictors of nutrient intakes in a large study of black and white men in the United States and, for trans fat intake, the effect of region was modified by race | More research is needed to understand if and how they play a role in the health disparities and chronic disease risks observed among racial groups and regions in the US. Also, clarifying the effects of race and region on dietary intakes may be helpful in making dietary recommendations and informing interventions for specific population subgroups. |
|  | **Niven 2014** | Australia | Cross-sectional | Secondary-school students (n 12 188) aged 12-17 years participating in the 2009-10 nassda | Adolescents | To assess the association between socio-economic position (SEP) and poor eating behaviours in a large representative sample of Australian secondaryschool students. | SES: Contextual area-level disadvantage | Sex, School year, Postcode of residence (rural vs urban + geographical influences) | FC: F/VSSBFast food | Students of lower-SEP areas were more likely to report low intake of vegetables and high frequency of consumption of sugar-sweetened beverages and fast food compared with students of high-SEP neighbourhoods. A positive SEP association was found for fruit consumption among female students. Those from lower-SEP areas were also more | Students of lower-SEP areas were more likely to report low intake of vegetables and high frequency of consumption of sugar-sweetened beverages and fast food compared with students of high-SEP neighbourhoods. A positive SEP association was found for fruit consumption among female students. Those from lower-SEP areas were also more | Reducing social inequalities in eating behaviours amongYoung people should be a key consideration of futurePreventive strategies |
|  | **Noonan 2018** | United Kingdom | Cohort Study | Ten thousand seven hundred thirty-six adolescents (5425 boys) | Adolescents | To (1) determine whether an income gradient to overweight and obesity exists in UK adolescents, and (2) examine associations between poverty, weight status, and dietary intake among adolescent girls and boys | SES: Income | Ethnicity and gender | FC: F/V, Sweetened drinks, Fast Food | Regular fruit and vegetable consumption was low, with only 30.9% and 37.6% of participants eating them daily, respectively. In contrast, 23.7% consumed sweetened drinks daily, and 68.6% weekly. Fast food was eaten daily by just 1.8% and weekly by 28.8%. Overall, girls had better diet quality than boys, but boys in poverty consumed more fruit, sweetened drinks, and fast food than girls in similar circumstances. Both boys and girls living in poverty reported higher intake of sweetened drinks and fast food, and lower intake of fruits and vegetables compared to their peers not living in poverty, with larger disparities noted among girls in weight status and dietary outcomes. | Poor dietary intake among adolescents living in poverty in this study is explained in most part by the economic constraints of living in poverty. A constrained family food budget is positively related to a high energy, low nutrient diet, and extensive research shows that few disadvantaged adolescents meet fruit and vegetable recommendations . | Government policy level interventions are required to reduce poverty inequalities in adolescent diet and obesity to prevent health disparities continuing into adulthood, which convey negative health and economic consequences. |
|  | **Park 2020** | Korea | Cross-sectional | Older adults aged ≥ 65 years (n = 3207) who participated in the KNHANES. Urban and rural areas classified the region and the Korean Healthy Eating Index (KHEI) assessed the diet quality. Personal factors that were related to diet quality included socio-demographic factors, health behaviors, and health conditions | Elderly | This study aimed to investigate whether there is an area difference on diet quality and the effect of personal factors on diet quality is different by areas among the Korean elderly population, while using the KNHANES data. | Rural/Urban | Age, sex, household type, marital status, education level, household income, job status, home ownership, beneficiaries of national basic livelihood, food assistance program participation, food security, smoking, alcohol drinking, walking exercise, nutritional education, eating with others, limited social activity due to disability, weight status, chronic disease, depression, and perceived poor oral health. | P: Korean Healthy Eating Index (KHEI) | This study found that the diet quality was different between urban and rural areas in the Korean elderly population, showing a higher mean of KHEI scores in urban areas than rural areas (67.3 for urban seniors, 63.6 for rural seniors, p < 0.001), and the regional difference was still significant, even after adjusting for the personal factors (p < 0.001). Different sets of personal factors were found to be significant that explain the diet quality of participants between areas, such as economic resources, walking exercise, and perceived oral health status in urban areas, and age and food insecurity in rural areas. | The differences in diet quality between rural and urban Korean elderly can be attributed to various factors. Firstly, poor food access in rural areas contributes to lower intake of essential foods like fruits, milk, and dairy products. Economic disparities also play a role, affecting food availability and accessibility. Moreover, reliance on farming or home gardening in rural areas limits food variety. In urban regions, economic resources such as household income significantly influence diet quality, while factors like walking exercise and perceived oral health also play a role. Community food environments, particularly in rural areas, impact diet quality, with limited food accessibility contributing to lower diet quality. | Tailored interventions considering both individual characteristics and local food environments are crucial to addressing regional dietary disparities and improving overall diet quality in older adults, especially in vulnerable rural areas. |
|  | **Parker 2020** | United States | Cross-sectional | Women in their third trimester (n 1322) with dietary history | Pregnant women | To examine differences in prenatal diet quality by socio-economic status (SES) and race/ethnicity | Multiple: race, income | Age, smoking, race and energy intake. | P: Alternative Healthy Eating Index for Pregnancy | High-income women had higher total (62·4 (SE 1·0)) and moderation component AHEI-P scores than middle-income (60·1 (SE 0·8), P = 0·02), low-income WIC participants (58·3 (SE 0·8), P < ·0001) and non-participants (58·9 (SE 0·9), P = 0·001). Nonhispanic Black participants had lower total (57·8 (SE 1·4)) and adequacy scores than Other races (i.e. Neither non-Hispanic Black nor White, 62·1 (SE 0·9), P = 0·02). | NA | Interventions are needed to increase the intake of adequacy components (particularly in NHB women) and decrease the intake of moderation components (particularly in low- and middle-income women and WIC participants) |
|  | **Patel 2020** | United Kingdom | Cross-sectional | Included 6416 adults (>18 year) (3741 women and 2675 men) included in the database. | Adults | To evaluate recent trends of the DASH score across socio-economic strata of the UK population, using education, occupation and income as proxies of the SEP. | SES: Education, occupation, income | Dietary assessment ethnic group  BMI | P: DASH | We found that the DASH score increased over time in all socio-economic groups in the UK; however, less educated individuals, those engaged in routine occupations and subjects with lower incomes had lower scores, indicating a persisting socio-economic gap. This gap was mainly driven by a lower intake of fruit, vegetables, whole grains, nuts, legumes and seeds. | Persisting socio-economic gap. | And calls for more effective promotion of healthy diet in the most disadvantaged individuals. |
|  | **Pitts 2015** | United States | Cross-sectional | To represent geographic diversity within NC, we selected counties in each of three distinct geographic regions of North Carolina: the Western mountains, Central piedmont, and Eastern coastal plain. Within each region, we selected one rural and one urban county in which to conduct a random-digit-dial (RDD) survey about farmers’ market shopping, similar to a previous RDD survey | Adults | To examine (1) associations between county-level zoning to support farmers’ market placement and county-level farmers’ market availability, rural/urban designation, percent African American residents, and percent of residents living below poverty and (2) individual-level associations between zoning to support farmers’ markets; fruit and vegetable consumption and body mass index (BMI) among a random sample of residents of six North Carolina (NC) counties. | Multiple: food environment, ethnicity, povery, residence characteristics | Age, race, sex, educational level, and county-level metro status | FC: F/V | Healthier food zoning in more urban areas , healthier food zoning in areas with less poverty. As the Healthy Outlet Zoning Score increased, there was an inverse association between shopping at farmers’ markets and BMI. The Healthy Outlet Zoning Scores were positively associated with self-reported fruit and vegetable consumption and inversely associated with BMI. | NA | Such studies are needed to inform future efforts to reduce health disparities, including interventions targeting zoning modifications. Changes to zoning related to the food environment may help improve food access and reduce health disparities, particularly among disadvantaged populations. |
|  | **Poti 2016** | United States | Cohort study | 656,184 household year-level observations (157,142 unique households) from 2000 through 2012. | Households | To examine the independent associations of race/ethnicity with highly processed and ready-to-eat (RTE) food purchases among US households | Ethnicity | Education, income, household composition, number of household members in each age and sex category, and geographic market. | FC: highly processed and ready to eat foods | Black households had significantly lower purchases of highly processed foods and RTE convenience foods and had higher purchases of basic processed foods, particularly cooking oils and sugar, foods requiring cooking/preparation and highly processed beverages .  Hispanics also had lower purchases of highly processed and RTE foods than whites. Blacks had CPG purchases with significantly higher median sugar and energy density , whereas Hispanics had purchases with lower saturated fat and energy density than whites. | Differences in the quality of home-prepared meals or cooking methods across populations have been suggested as explanations of why foods cooked at home may not necessarily be more healthful than convenience foods | Findings have major implications for the foods and behaviors that future interventions or policy might target to improve racial/ethnic disparities. Further investigation is warranted to examine how purchasing patterns of basic processed foods used in cooking among vulnerable populations may contribute to disparities in diet and health. |
|  | **Raffensperger 2010** | United States | Cross-sectional | African American and white adults ages 30-64 residing in 12 predefined census tracts in Baltimore | Adults | To examine effects of race and predictors of socioeconomic status (SES) on nutrient-based diet quality and their contribution to health disparities in an urban population of low SES. | Ethnicity and SES indicators | Sex, race, PIR,education, and income | P: HEI, MAR, NARNC: Energy, macronutrients and fibre consumption | Sex, age, education, PIR, and income were statistically significant predictors of diet quality for African Americans, while sex, education, and smoking status were statistically significant for whites. African Americans had lower MAR scores than whites (76.4 vs. 79.1). Whites had significantly higher NAR scores for thiamin, riboflavin, folate, B12, vitamins A and E, magnesium, copper, zinc, and calcium, while African Americans had higher vitamin C scores. | The findings of this study indicate education was an important, if not the most important predictor of nutrient-based diet quality in the HANDLS sample. Lower diet quality among African Americans examined in the HANDLS study may reflect cultural differences in selection and preparation of foods. | Culturally appropriate nutrition education to both African Americans and whites, particularly messages aimed at individuals with less than a high school education would be beneficial to improving diet quality in urban areas of low SES. |
|  | **Ranjit 2014** | United States | Cross-sectional | 2502 children (51.8% female; mean age: 13.9 years) | Children Adolescents | To examine racial and ethnic differences in the home food environment and healthy eating | Ethnicity | Age, sex, language spoken at home, and body mass index z-score, home food environment score, SES | P: Healthy diet Unhealthy diet FC: F/V Healthy beverages Unhealthy beverages SSB snacks desserts | White children had significantly better HFEsthan Hispanic and black children with greater availability and accessibility of healthy foods. Adjusting for a healthy HFE reduced disparities in consumption of healthy foods but not in consumption of unhealthy foods | Food home environment is linked to purchase power (conditioning healthy food choices). Moreover, both cultural factors and unmeasured SES markers could account for some of these racial and ethnic differences, including psychosocial stress  and sociocultural influences on food preferences, shopping practices, time allocation, child-rearing strategies, and priorities are all potentially important determinants of home food availability. | Improving the home food environment (ie, increasing access and availability of healthy foods) could increase the consumption of healthy foods among racial and ethnic minority adolescents |
|  | **Rashid 2018** | The Netherlands | Cross-sectional | We analysed 2769 validated Food Frequency Questionnaires filled in by mothers of children (5.7 ± 0.5y) in the Amsterdam Born Children and their Development (ABCD) cohort. | Children | We derived dietary patterns at age 5 and determined whether ethnicity and SES were both related to these dietary patterns. | Ethnicity | SES, age, gender and maternal age | P: Post-hoc dietary patterns | Principal Component Analysis identified 4 dietary patterns: a snacking, full-fat, meat and healthy dietary pattern, explaining 21% of the variation in dietary intake. Ethnicity was related to the dietary pattern scores (p < 0.01): non-Dutch children scored high on snacking and healthy pattern, whereas Turkish children scored high on full-fat and Surinamese children on the meat pattern. SES was related to the snacking, full-fat and meat patterns (p < 0.01): low SES children scored high on the snacking and meat pattern and low on the full-fat pattern. | NA | Future studies could analyse the explanatory factors in early childhood contributing to these (differences in) dietary choices and the possible relationships these dietary patterns may have with weight development and health inequalities in later childhood. |
|  | **Rouche 2019** | Belgium | Cross-sectional | In total, 19,172 school adolescents aged 10–19 years were included in analyses. | Adolescents | To estimate socioeconomic disparities in dietary habits of school adolescents from different migration backgrounds. | Ethnicity: Migrant background | Food Frequency Questionnaire Family affluence School region | FC: Main food groups consumption | Multilevel multiple binary and multinomial logistic regressions were performed, stratified by migration status (natives, 2nd- and 1st-generation immigrants). Overall, immigrants more frequently consumed both healthy and unhealthy foods. Indeed, 32.4% of 1st-generation immigrants, 26.5% of 2nd-generation immigrants, and 16.7% of natives consumed fish ≥two days a week. Compared to those having a high family affluence scale (FAS), adolescents with a low FAS were more likely to consume chips and fries ≥once a day (vs. <once a day: Natives arrr = 1.39 (95%CI: 1.12–1.73); NS in immigrants). Immigrants at schools in Flanders were less likely than those in Brussels to consume sugar-sweetened beverages 2–6 days a week (vs. ≤once a week: Natives arrr = 1.86 (95%CI: 1.32–2.62); 2nd-generation immigrants arrr = 1.52 (1.11–2.09); NS in 1st-generation immigrants). | The migration gradient observed here underlines a process of acculturation. Narrower socioeconomic disparities in immigrant dietary habits compared with natives suggest that such habits are primarily defined by culture of origin | Nutrition interventions should thus include cultural components of dietary habits. |
|  | **Stowers 2020** | United States | Cross-sectional | 4305 participants, reached through Amazon Mechanical Turk (mturk) (38% Male, 49% lower income, 67% non-hispanic white) | Adults | The study explores whether living in a perceived food desert and food swamp was more likely to be reported by lower-income or racial and ethnic minority individuals, and if such perceptions are related to lower-quality diets, higher weight, and worse reported health. | Ethnicity and residence characteristics | Family income, race/ethnicity, education level, current family structure, geographic area, car ownership, gender, region of the country, and age | P: Diet quality composite score | Non-Hispanic, Black participants (N = 954) were most likely to report living in a food swamp. In the full and White subsamples (N = 2912), the perception of residing in a food swamp/desert was associated with less-healthful self-reported dietary habits overall. For non-Hispanic Blacks, regression results also showed that residents of perceived food swamp areas (OR = 0.66, p < 0.01, 95% CI (0.51, 0.86)) had a lower diet quality than those not living in a food swamp/food desert area. | Racial seggregation | More policy efforts that address the disparities in access to unhealthy versus healthy food retail outlets across neighborhoods are needed to limit the negative impacts on nutrition and health-related outcomes. |
|  | **Eagle 2012** | United States | Cross-sectional | 09,634 Massachusetts children | Children | Define the association between childhood obesity and household income and how household income and childhood behaviors promote childhood obesity. | SES: area deprivation | None | FC: F/V, meat, french fries, fried foods, and milk | The prevalence of overweight/obese children rises in communities with lower household income. Children residing in lower income communities exhibit poorer dietary and physical activity behaviors, which affect obesity. | -Nutritional knowledge in households, parents' education 1. Child's health status involves a complex interplay between individual factors, social factors, environmental factors, and a child's ultimate selection (within their range of choices) of nutrient consumption and energy expenditure 2. Access to fast foods, poorer access to fresh fruits and vegetables, and poor access to recreational parks and both recreational and school-based exercise programs were very likely to play an important role in promoting childhood obesity in communities with lower mean family income. 3. The association between household income and childhood obesity also relates to parents. Single-parent households, on average, have lower income. Similarly, educational level of parent(s) directly relate to emphasis on healthy behaviors in and outside the home. | Encouraging parents and children to eat more healthily or to make healthy dietary choices and exercise more regularly seems logical and doable. However, a deeper examination into this national epidemic leads one to appreciate that the underpinnings to childhood obesity are remarkably complex, and the solutions are likely to be equally if not more complex. |
|  | **Thomson 2020** | United States | Cross-sectional | 5050 men (31.3% NHB, 68.7% NHW) NHANES | Children | To describe and compare the diet quality and nutrient intake of NHB and non-Hispanic white (NHW) men in the United States. | Ethnicity | Day of the week, age range, education level, poverty level, marital status, number of household members, and body mass index. | P: HEI | After adjusting for sociodemographic measures, NHB and NHW men had similar diet quality (P = .59). Compared with NHW men, NHB men had lower odds of meeting recommendations for dietary fiber and cholesterol intake and higher odds of meeting recommendations for saturated fat and sodium intake | Education and poverty level may be important contributors to racial/ethnic disparities in dietary intake often observed among men, which may influence cooking practices, household structure, and cultural attitudes toward healthy eating, among others | NA |
|  | **Van Ansem 2014** | The Netherlands | Cross-sectional | 1318 parent-child dyads from the INPACT study | Children + parent dyads | 1) to investigate the association between maternal educational level and healthy eating behaviour of 11-year-old children (fruit, vegetables and breakfast consumption), and 2) to examine whether factors in the home food environment (parental intake of fruit, vegetables and breakfast; rules about fruit and vegetables and home availability of fruit and vegetables) mediate these associations | SES: Education | Age, gender, ethnicity and body mass index (BMI) of the child | FC: F/V  O: Breakfast consumption | Children of mothers with a high educational level consumed more pieces of fruit per day (B = 0.13, 95% CI: 0.04-0.22), more grams of vegetables per day (B = 23.81, 95% CI = 14.93-32.69) and were more likely to have breakfast on a daily basis (OR = 2.97, 95% CI: 1.38-6.39) than children of mothers with a low educational level. Home availability, food consumption rules and parental consumption mediated the association between maternal education level and children’s fruit and vegetable consumption. Parental breakfast consumption mediated the association between maternal education level and children’s breakfast consumption. | Parental education shapes their behavior which, in turn, influences that of the children through food environment and rules | Targeting parental nutritional knowledge (especially among those with a low educational level) may be an effective way to improve the home food environment. Besides interventions that aim at the importance of family involvement, also multiple-setting interventions are effective in changing children's dietary behaviour. |
|  | **Vankim 2012** | United States | Cross-sectional | 1201 college students | University students | To explore weight, weight behaviors, and tobacco and alcohol use among emerging adults by parental education and financial strain. | SES: Education, income | Race/ethnicity, gender, employment, relationship status, number of children, year in school, and self-perception of being an adult | FC: F/V , Fast Food | Low parental education was associated with lower fruit/vegetable consumption and more fast food | NA |  |
|  | **Vankim2019** | United States | Cross-sectional | 12,880; aged 10 to 23 years | Children Adolescents | To examine sexual orientation and gender expression differences in diet quality and eating habits from adolescence to young adulthood | Sexual orientation | Age, race/ethnicity, and region of residence | P: Alternative HEI O: Breakfast consumption, Family dinners | "Gender-nonconforming" males had significantly higher diet quality scores than "very gender-conforming" males (P<0.05). Diet quality scores did not differ by gender expression among females. "Mostly heterosexual" females and gay males had higher diet quality scores than their same-sex completely heterosexual counterparts (P<0.05). Adjustment for mother's diet quality scores attenuated effects, except for gay males (P<0.05). "Gender-nonconforming" females were less likely to consume breakfast than "very gender-conforming" females (P<0.05). Similar results were found for "mostly heterosexual" and bisexual compared to completely heterosexual females. There were no gender expression or sexual orientation differences in family dinners among males and females. | - social perceptions of “masculinity” on various aspects of eating habits and diets (healthy eating being perceived as inherently feminine behavior) | - diet interventions targeted to men, may want to consider incorporating intervention elements that challenge perceptions of healthy eating as inherently feminine or particular food items as reinforcing masculine ideals. |
|  | **Wang 2016** | United States | Cross-sectional | 2669 women | Adults | To answer whether: (i) race/ethnicity, individual SES and neighbourhood SES have independent effects on women's fruit and vegetable consumption (FVC); (ii) SES modifies the effects of race/ethnicity on FVC; and (iii) nativity modifies the effect of Latina ethnicity on FVC. | Ethnicity, SES | Age (20–29 years; 30–39 years; ≥40 years), marital status (previously or never married; married or living together), race/ethnicity (from the birth certificate: non-Hispanic African American; non-Hispanic Asian or Pacific Islander; Latina; non-Hispanic White), country of birth (US-born; immigrant (for Latinas only)), educational attainment (less than high school; high-school graduate/GED; some college; college graduate (where GED is General Educational Development)), income (annual family income, in increments of the federal poverty level: ≤100 %; 101–200 %; 201–300 %; 301–400 %; >401%; missing) and longitudinal neighbourhood poverty | FC: F/V | In adjusted models, race/ethnicity, education and income were independently associated with FVC, but not neighbourhood poverty. Women of colour, high-school graduates and women with incomes at 301-400 % of the federal poverty level were at higher odds of LOWFV compared with non-Hispanic Whites, college graduates and those with incomes >400 % of the federal poverty level. Little evidence for interactions between race/ethnicity and individual or neighbourhood SES was found; similar patterns were observed for immigrant and US-born Latinas | NA | Addressing the dietary needs of lower-SES communities requires multilevel interventions that simultaneously provide culturally tailored nutrition education and address the physical and economic accessibility of culturally acceptable fruits and vegetables |
|  | **Welker 2018** | United States | Cross-sectional | 600 children 24-47.9 mo old from the FITS 2016 study (47% male, 65%NH white, 18% NH black, 14% hispanic, 2.8% other) | Children | To analyze the current food and beverage intake patterns of 2- and 3-y-olds (i.e., children aged 24–35.9 mo and 36–47.9 mo, respectively), and how these food and beverage intake patterns differ among racial/ethnic groups | Ethnicity | None | FC: Several food groups NC: energy from food groups (mean kilocalories per consumer). | Significant differences (although not corrected for type 1 error) were found for dairy products, meat products and ssbs | Cost of diet, physical access, targeted marketing | Individual-, community-, and policy-level shifts are needed to improve children’s diets. Examples of strategies may include exposing children to a variety of healthful foods at a young age, in addition to repeated exposure to those foods; role modeling of healthy eating by parents and caregivers; improving the food environment in child care settings; incentivizing the purchase of fruit and vegetables within SNAP; and continuing to review and improve the WIC food package to ensure that it meets the nutritional needs of women, infants, and young children. |
|  | **Wilson 2021** | Australia | Cohort study | 1482 participants who were 10–15 years of age in 1985 | Children  Adolescents | To examine associations between three SES mobility variables (area disadvantage, education, occupation) and adult diet quality | SES: area disadvantage, education, occupation | Language spoken at home (categorized as English, European languages, and other languages), adulthood measures (age at CDAH follow-up and marital status), physical activity measures (MET minutes and total weekly MET hours), residential remoteness, BMI (weight, height, and calculated BMI), and usual energy intake per day derived from the FFQ. | P: Dietary Guideline Index | Area-level SES mobility was not associated with diet quality. Compared with stable high (university) education, stable low (school only) was associated with lower DGI scores as was downward educational mobility (participant’s education lower than their parents) and stable intermediate (vocational) education among males Compared with stable high (professional/managerial) occupation, stable low (manual/out of workforce) males, and participants with downward occupation mobility ( had lower DGI scores. Intergenerational low education and occupation, and downward educational and occupational mobility, were associated with poor adult diet quality. | Peer/social influence or higher income to support better dietary variety and choices due to health literacy, skills to seek out help, interpret and apply information, nutritional knowledge, cost of diet. | Strategies to both improve formal education opportunities and implement health education campaigns, particularly targeted to those from socio-economically disadvantaged backgrounds. These strategies could be complemented by government policies such as providing adequate welfare payments or subsidising healthy foods to support affordability of nutritious diets |
|  | **Woolf 2020** | United States | Cross-sectional | 997 4th to 5th grades who completed the School Nutrition and Physical Activity Survey | Children | To investigate differences in eating behavior of youth by race/ethnicity and socioeconomic status. | Ethnicity, income (free-paid lunch) | Sex, school | FC: Main food groups consumption O: consumption of breakfast, consumption of an evening meal, and eating at a restaurant on the previous day | Minority youth were less likely to consume healthy proteins and more likely to eat at a restaurant compared to white youth. Lower socioeconomic status youth were less likely to eat an evening meal compared to higher socioeconomic status youth. | Food accessibility (in the neighborhoods and at the schools), d skills or time for food preparation and food insecurity | . This study provides several implications for actions schools and their partners can take: • Account for school environmental factors in research and interventions • Educate teachers on nutrition education implementation in the classroom  • Expand research and action on nutrition to involve families and siblings • Emphasize time-specific activities and familial influences • Educate and involve family during the intervention period • Schools should utilize an intersectional approach during research • Investigate cultural, religious, and racial/ethnic difference |
|  | **Zagorsky 2017** | United States | Longitudinal/Cross-sectional | 8136 adults from the 2008, 2010, and 2012 waves of the National Longitudinal Survey of Youth (NLSY79) | Adults | To examine whether there is an SES gradient in one specific aspect of nutrition: fast-food consumption | SES: Income & wealth |  | FC: Fast food | There is no clear income or wealth gradient, with individuals from various socioeconomic backgrounds consuming fast food. Moreover, contrary to stereotypes, those in the lowest income quintile consumed fast food less frequently than those in higher quintiles. Becoming richer or poorer does not lead adults to change their fast-food consumption. | NA | Targeting central cities and the South could be beneficial when implementing policies to reduce adult fast-food intake |
|  | **Zarnowiecki 2014** | Australia | Cross sectional | 395 9-13 years and their parents | Children | To determine whether the associations between personal and environmental variables and children's fruit and vegetable intake, and healthy dietary behaviours are moderated by SEP | Multiple: Residence Characteristics, Occupations, Employment, Educational Status, Income | Socio-economic moderators, neighbourhood SEP, child age, marital status and mother’s age | FC: F/V | Fruit and vegetable intake and healthy behaviours were predicted by self-efficacy, attitudes and a supportive home environment. For girls, only the associations of self-efficacy with healthy behaviours were moderated by occupation. For boys, income moderated the associations of fruit and vegetable intake with attitudes, and healthy behaviours with supportive home environments. Occupation and employment moderated the associations of boys' family environments and fruit intake, and attitudes with healthy behaviours. | Reducing socio-economic disparities in children's healthy dietary intake may be more successfully achieved by tailoring health promotion policies and interventions according to variables that moderate the relationships between dietary intake and SEP. | Helping parents to create supportive home environments from which children may develop more positive attitudes and self-efficacy for healthy eating may contribute to improvements in dietary intake among children of low SEP |

| **Supplementary table 2. Quality assessment of the sampled studies, based on the Mixed Methods Appraisal Tool (MMAT).** | | | | | | | |
| --- | --- | --- | --- | --- | --- | --- | --- |
|  | **SCREENING QUESTIONS** | | **3. NON-RANDOMIZED STUDIES** | | | | |
| Study | S1. Are there clear research questions? | S2. Do the collected data allow to address the research questions? | 3.1. Are the participants representative of the target population? **4.1. Is the sampling strategy relevant to address the research question?** | 3.2. Are measurements appropriate regarding both the outcome and intervention (or exposure)? **4.2. Is the sample representative of the target population?** | 3.3. Are there complete outcome data?   **4.3. Are the measurements appropriate?** | 3.4. Are the confounders accounted for in the design and analysis? **4.4. Is the risk of nonresponse bias low?** | 3.5. During the study period, is the intervention administered (or exposure occurred) as intended? **4.5. Is the statistical analysis appropriate to answer the research question?** |
| Aggarval 2011 | Yes | Yes | Yes | Yes | Yes | Yes | Yes |
| Assari 2020 | Yes | Yes | Yes | No | Yes | Yes | Yes |
| Bayram 2018 | Yes | Yes | Yes | NA | Yes | Yes | Yes |
| Bekelman 2020 | Yes | Yes | Yes | Yes | Yes | Yes | Yes |
| Bekelman 2020 | Yes | Yes | Yes | Yes | Yes | Yes | Yes |
| Bolt Evensen 2018 | Yes | Yes | Yes | No | Yes | Yes | Yes |
| Brunst 2013 | Yes | Yes | Yes | No | Yes | Yes | Yes |
| Carroll-Scott 2013 | Yes | Yes | Yes | Yes | Yes | Yes | Yes |
| Cavaliere 2019 | Yes | Yes | Yes | Yes | Yes | Yes | Yes |
| Chen Ji 2014 | Yes | Yes | Yes | NA | Yes | Yes | Yes |
| Chzhen 2018 | Yes | Yes | Yes | Yes | Yes | Yes | Yes |
| Esquius 2021 | Yes | Yes | Yes | Yes | Yes | Yes | Yes |
| Friis 2016 | Yes | Yes | Yes | Yes | Yes | Yes | Yes |
| Guerrero 2015 | Yes | Yes | Yes | Yes | Yes | Yes | Yes |
| Haughton 2016 | Yes | Yes | Yes | Yes | Yes | Yes | Yes |
| Hauschildt 2019 | Yes | Yes | Yes | NA | Yes | Yes | Yes |
| Highland 2016 | Yes | Yes | Yes | NA | Yes | Yes | Yes |
| Kurotani 2021 | Yes | Yes | Yes | Yes | Yes | Yes | Yes |
| Larson 2015 | Yes | Yes | Yes | Yes | Yes | Yes | Yes |
| Lee 2019 | Yes | Yes | Yes | Yes | Yes | Yes | Yes |
| Lee 2020 | Yes | Yes | Yes | Yes | Yes | Yes | Yes |
| Liu 2021 | Yes | Yes | Yes | Yes | Yes | Yes | Yes |
| Livingstone 2017 | Yes | Yes | Yes | Yes | Yes | Yes | Yes |
| Luk 2018 | Yes | Yes | Yes | Yes | Yes | Yes | Yes |
| Lutfiyya 2012 | Yes | Yes | Yes | Yes | Yes | Yes | Yes |
| Manyanga 2017 | Yes | Yes | Yes | Yes | Yes | Yes | Yes |
| Martin 2017 | Yes | Yes | Yes | Yes | Yes | Yes | Yes |
| Martin 2017 | Yes | Yes | Yes | Yes | Yes | No | Yes |
| Martínez-Martínez 2020 | Yes | Yes | Yes | Yes | Yes | Yes | NA |
| Maruyama 2017 | Yes | Yes | Yes | Yes | Yes | Yes | Yes |
| McCartney 2013 | Yes | Yes | Yes | Yes | Yes | Yes | Yes |
| Méjean 2016 | Yes | Yes | Yes | Yes | Yes | Yes | Yes |
| Mendez 2019 | Yes | Yes | Yes | Yes | Yes | Yes | Yes |
| Miura 2012 | Yes | Yes | Yes | Yes | Yes | Yes | Yes |
| Moore 2014 | Yes | Yes | Yes | Yes | Yes | Yes | Yes |
| Morgan 2021 | Yes | Yes | Yes | Yes | Yes | Yes | Yes |
| Newby 2010 | Yes | Yes | Yes | Yes | Yes | Yes | NA |
| Niven 2014 | Yes | Yes | Yes | Yes | Yes | Yes | NA |
| Noonan 2018 | Yes | Yes | Yes | Yes | Yes | Yes | NA |
| Park 2020 | Yes | Yes | Yes | Yes | Yes | Yes | NA |
| Parker 2020 | Yes | Yes | Yes | Yes | Yes | Yes | NA |
| Patel 2020 | Yes | Yes | Yes | Yes |  | Yes | NA |
| Pitts 2015 | Yes | Yes | Yes | Yes | Yes | No | Can't tell |
| Poti 2016 | Yes | Yes | Yes | Yes | Yes | Yes | Yes |
| Raffensperger 2010 | Yes | Yes | Yes | Yes | Yes | Yes | Yes |
| Ranjit 2014 | Yes | Yes | Can't tell | Yes | Yes | Yes | NA |
| Rashid 2018 | Yes | Yes | Yes | Yes | Yes | Yes | Yes |
| Rouche 2019 | Yes | Yes | Yes | Yes | Yes | Can't tell | Yes |
| Stowers 2020 | Yes | Yes | No | Yes | Yes | Can't tell | Yes |
| Eagle 2012 | Yes | Yes | Yes | Yes | Yes | Yes | NA |
| Thomson 2020 | Yes | Yes | Yes | Yes | Yes | Yes | Yes |
| van Ansem 2014 | Yes | Yes | Yes | Yes | Yes | Yes | Yes |
| VanKim 2012 | Yes | Yes | Yes | Can't tell | Yes | Yes | Yes |
| VanKim2019 | Yes | Yes | Yes | Yes | Yes | Yes | Yes |
| Wang 2016 | Yes | Yes | Yes | Yes | Yes | Yes | Yes |
| Welker 2018 | Yes | Yes | Yes | Yes | Yes | Yes | Yes |
| Wilson 2021 | Yes | Yes | Yes | Yes | Yes | Yes | Yes |
| Woolf 2020 | Yes | Yes | Yes | Yes | Yes | Yes | Yes |
| Zagorsky 2017 | Yes | Yes | Yes | Yes | Yes | Yes | Yes |
| Zarnowiecki 2014 | Yes | Yes | Yes | Yes | Yes | Yes | Yes |
